# RNA-Seq and differential gene expression analysis in *Temora stylifera* copepod females with contrasting non-feeding nauplii survival rates: an environmental transcriptomics study

**DOI:** 10.1186/s12864-020-07112-w

**Published:** 2020-10-06

**Authors:** Ennio Russo, Chiara Lauritano, Giuliana d’Ippolito, Angelo Fontana, Diana Sarno, Eric von Elert, Adrianna Ianora, Ylenia Carotenuto

**Affiliations:** 1 Stazione Zoologica Anton Dohrn, Villa Comunale, 80121 Naples, Italy; 2 Consiglio Nazionale delle Ricerche, Institute of Biomolecular Chemistry, Via Campi Flegrei 34, 80078 Pozzuoli, Italy; 3 Universität zu Köln, Aquatic Chemical Ecology Group, Zülpicher Straβe 47b, D-50674 Cologne, Germany

**Keywords:** De novo transcriptome assembly, Differential gene expression, Copepod, *Temora stylifera*, Maternal effects, Reproduction, Environmental transcriptomics

## Abstract

**Background:**

Copepods are fundamental components of pelagic food webs, but reports on how molecular responses link to reproductive success in natural populations are still scarce. We present a de novo transcriptome assembly and differential expression (DE) analysis in *Temora stylifera* females collected in the Gulf of Naples, Mediterranean Sea, where this copepod dominates the zooplankton community. High-Throughput RNA-Sequencing and DE analysis were performed from adult females collected on consecutive weeks (May 23rd and 30th 2017), because opposite naupliar survival rates were observed. We aimed at detecting key genes that may have influenced copepod reproductive potential in natural populations and whose expression was potentially affected by phytoplankton-derived oxylipins, lipoxygenase-derived products strongly impacting copepod naupliar survival.

**Results:**

On the two sampling dates, temperature, salinity, pH and oxygen remained stable, while variations in phytoplankton cell concentration, oxylipin concentration and oxylipin-per-diatom-cell production were observed. *T. stylifera* naupliar survival was 25% on May 23rd and 93% on May 30th. De novo assembly generated 268,665 transcripts (isoforms) and 120,749 unique ‘Trinity predicted genes’ (unigenes), of which 50% were functionally annotated. Out of the 331 transcript isoforms differentially expressed between the two sampling dates, 119 sequences were functionally annotated (58 up- and 61 down-regulated). Among predicted genes (unigenes), 144 sequences were differentially expressed and 31 (6 up-regulated and 25 down-regulated) were functionally annotated. Most of the significantly down-regulated unigenes and isoforms were *A5 Putative Odorant Binding Protein* (*Obp*). Other differentially expressed sequences (isoforms and unigenes) related to developmental metabolic processes, protein ubiquitination, response to stress, oxidation-reduction reactions and hydrolase activities. DE analysis was validated through Real Time-quantitative PCR of 9 unigenes and 3 isoforms.

**Conclusions:**

Differential expression of sequences involved in signal detection and transduction, cell differentiation and development offered a functional interpretation to the maternally-mediated low naupliar survival rates observed in samples collected on May 23rd. Down-regulation of *A5 Obp* along with higher quantities of oxylipins-per-litre and oxylipins-per-diatom-cell observed on May 23rd could suggest oxylipin-mediated impairment of naupliar survival in natural populations of *T. stylifera*. Our results may help identify biomarker genes explaining variations in copepod reproductive responses at a molecular level.

## Background

Among zooplankton, marine and freshwater copepods represent one of the most morphologically and functionally diverse groups [1], playing a central role in food web dynamics and biogeochemical cycles [2]. In this perspective, assessment of biotic and abiotic factors influencing copepod populations can be of primary importance to understand marine pelagic food web functioning. Phytoplankton-derived oxylipins potentially represent key factors affecting wild copepod populations [3]. These molecules are end products of well characterized enzymatic pathways activated after cell wounding, starting from lipolytic release of free fatty acids (FFAs) from complex lipids [4–6] and proceeding through oxygenation of FFAs by lipoxygenases (LOX) [5, 7–16].

In the last two decades, extensive evidence was reported about impaired reproductive success in copepod females fed oxylipin producing diatoms, which led to detrimental effects on egg production rates, egg hatching and survival of non-feeding nauplii (NI/NII) through a maternal effect [9, 17–25]. Since 2011, a number of studies have started to inspect the effects of oxylipin producing diatoms on the molecular responses of copepod females, evaluating variations in the quantitative expression of selected genes of interest [26–31] and applying a suppression subtractive library approach to gain insight into copepod responses at a transcriptomic level [32]. Very recently, the de novo assembled transcriptome of copepod females feeding on oxylipin-producing diatoms has been also generated [33].

Variations in copepod egg production, hatching success and naupliar survival in response to phytoplankton abundance and composition have been investigated in several copepod species through field surveys [3, 22, 34–42], but information about the molecular responses of adult females from natural populations are still limited to the Northern Ariatic Sea [35].

In the present survey, we investigated the molecular responses of adult females of the calanoid copepod *Temora stylifera* from the Gulf of Naples (GoN), where it dominates the autumnal copepod community [40, 43–46]. The GoN has been traditionally described as an oligotrophic basin showing low phytoplankton densities and consequent low oxylipin concentrations. However, we recently showed that high oxylipin-per-litre concentration and oxylipin-per-diatom-cell productions seasonally occur in this area [47]. Several studies have already investigated the population dynamics of *T. stylifera* in th GoN, exploring whether abiotic factors and life-history traits could explain the marked seasonality of this copepod in the area [40, 44, 48]. However, no genomic and transcriptomic information are available for this species.

Through a High-Throughput Sequencing approach, we generated a de novo assembled transcriptome of adult *T. stylifera* females. We also performed a Differential Expression (DE) analysis between specimens collected on two consecutive weeks (the 23rd and the 30th of May 2017), when early non-feeding nauplii with opposite survival rates (25% vs 93%, respectively) were laid.

Analyses of de novo assembled transcriptomes were reported to explore the biosynthetic pathways of gaseous signals [49], the enzymatic processes leading to hormone biosynthesis [50], reproductive processes [51, 52], including diapause [53–56] as well as responses to stress [57, 58] and phycotoxins [59] in several pelagic copepod species. Our results offer the opportunity to understand if molecular responses of *T. stylifera* females from natural populations can help to better explain different naupliar survival rates in relation to environmental (temperature, salinity, pH and oxygen), biological (phytoplankton abundance and composition) and biochemical (phytoplankton-derived oxylipins) variables [47].

## Results

### Environmental, chemical and biological variables

Information about abiotic (temperature, salinity, pH and oxygen), phytoplankton and oxylipin variations on the two selected dates are reported in Table 1. Abiotic variables did not show wide variations between the two sampling dates. In contrast, more pronounced variations were detected in phytoplankton community abundance and composition, when considering major phytoplankton groups (i.e. coccolithophores, dinoflagellates and phytoflagellates < 10 μm) as well as the most abundant diatom genera (i.e. *Chaetoceros*, *Skeletonema*, *Leptocylindrus*, *Pseudo-nitzschia*, *Thalassiosira* and the mixed group “other diatoms”). In general, phytoplankton was less concentrated on the 23rd of May (14.74 10^6^ cells/L) than the 30th of May (17.64 10^6^ cells/L). In particular, coccolithophores, dinoflagellates and the diatom genus *Chaetoceros* occurred at higher concentrations on the 23rd of May than the 30th, while higher densities of phytoflagellates < 10 μm and of the “other diatoms” group were observed on the 30th of May than the 23rd.

**Table 1:** Abiotic variables, phytoplankton composition and oxylipins

Variables	May 23rd	May 30th	Unit
*Environmental variables*			
Oxygen	5.36	5.32	**mg/m**^**3**^
pH	8.14	8.15	**0–14**
Salinity	37.65	37.88	**PSU**
Temperature	20.74	21.18	**°C**
*Phytoplankton*			
Coccolithophores	221,568	199,411	**cells/L**
Dinoflagellates	652,637	351,862	
Phytoflagellates < 10 μm	8,973,507	10,701,737	
*Chaetoceros*	2,149,211	1,191,802	
*Leptocylindrus*	1,440,193	2,104,897	
*Skeletonema*	110,784	332,352	
*Pseudo-nitzshia*	625,186	1,528,819	
*Thalassiosira*	0	354,509	
Other diatoms	562,710	874,964	
**TOTAL**	**14,735,796**	**17,640,353**	
*Oxylipins/L*			
HDoHE	85.16	34.25	**ng/L**
EHDPE	4.89	8.73	
HEPE	92.27	97.19	
EHETE	20.5	26.48	
HHTrE	0	2.13	
EHHDE	0	4.69	
**TOTAL**	**202.82**	**173.47**	
*Oxylipins/diatom cell*			
HDoHE-cell	17.42	5.36	**fg/cell**
EHDPE-cell	1.00	1.37	
HEPE-cell	18.88	15.22	
EHETE-cell	4.19	4.15	
HHTrE -cell	0	0.73	
EHHDE-cell	0	0.33	
**TOTAL-cell**	**41.49**	**27.16**	

List of the measured environmental variables, phytoplankton abundance and composition, oxylipin-per litre (Oxylipins/L) concentration and oxylipin-per-diatom-cell (Oxylipins/diatom-cell) production measured at LTER-MC on the 23rd and the 30th of May 2017. Measure units are shown. Major phytoplankton groups and main diatom genera are reported. Oxylipin species: HDoHE = hydorxy-docosahexaenoic acid, EHDPE = epoxy-hydroxy-docosapentaenoic acid, HEPE = hydroxy-eicosapentaenoic acid, EHETE = epoxy-hydroxy-eicosatetraenoic acid, HHTrE = hydroxy-hexadecatrienoic acid, EHHDE = epoxy-hydroxy-hexadecadienoic acid

Similarly, oxylipin concentrations were also higher on the 23rd (202.82 ng/L) than the 30th (173.47 ng/L) of May. Also, oxylipin-per-cell production was higher on the 23rd of May (41.49 fg/diatom-cell) than the 30th (27.16 fg/diatom-cell).

**Fig. 1:**
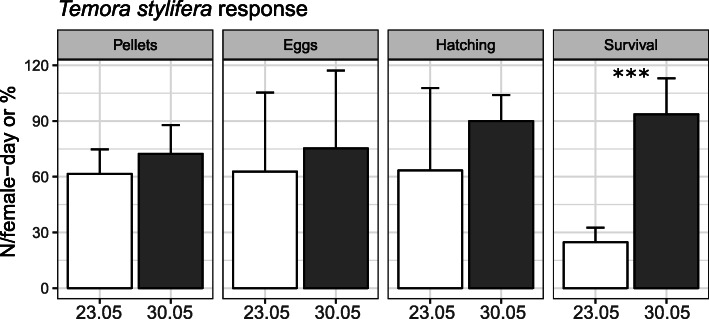
*Temora stylifera* responses. Average daily faecal pellet and egg production (N per female per day) measured in adult females as well as average egg hatching success and NI naupliar survival (%) for the two sampling dates (May 23rd and May 30th 2017). Differences in production or percentage were analysed through t-test (95% confidence interval). Significance level: *** < 0.001

T-test results demonstrated that early-life history traits estimated for *T. stylifera* on the two sampling dates (May 23rd and 30th) differed significantly in terms of survival rates of NI nauplii (25 and 93% of survival, respectively, *p* < 0.001, *N* = 15, Fig. 1). By contrast, non-significant differences (*p* > 0.01, N = 15) were observed in the number of faecal pellets (an indirect measure of feeding rates) (61.6 ± 3.39 and 72.35 ± 4.13 pellets per female per day, respectively), the number of spawned eggs (62.8 ± 11 and 75.36 ± 11.19 eggs per female per day, respectively) and the percentage of egg hatching success (63.4 ± 12.3% and 89.95 ± 4.05%, respectively).

### De novo *assembly and functional annotation of Temora stylifera transcriptome*

Total RNA extracted from pools of *T. stylifera* females collected on May 23rd and 30th had an average concentration of 232.7 ng/μl, with RIN = 10 and 260/280 as well as 260/230 ratios ~ 2. Illumina-based RNA-Seq generated a total of ~ 132 million reads, after quality cleaning. The same number of reads was achieved for both the forward and the reverse cDNA filaments, supporting consistency in the sequencing output. Raw reads are stored into the NCBI Sequence Read Archive database under accession numbers PRJNA632714. The de novo assembly made with Trinity on high quality reads generated 268,665 transcripts (isoforms) (average length of 517.6 bp, N_50_ = 665), and contained 120,749 ‘Trinity predicted genes’ (unigenes), i.e. non-redundant transcripts with unique TR#_c#_g# identifiers (Additional file 1: Table S1). Both the full (transcript isoforms) and the reference (unigenes) transcriptome, the latter consisting of the longest transcript isoform of each predicted gene, were processed for functional annotation. However, detailed description of annotation results is here provided only for the reference transcriptome.

Blast2Go mapping outputs indicated that almost 10% of the matching unigenes showed very high homology (0 < E-value< 10^− 100^) to similar sequences in the non-redundant protein database. Overall, more than 42% of the sequences showed high probability of homology (0 < E-value< 10^− 30^). Similarity values, which express the similarity percentage between the de novo assembled sequence and proteins in the non-redundant database, highlighted that a low fraction (1.7%) of the total unigenes were almost identical (similarity between 95 and 100%), while 76.1% of the sequences had a similarity ranging from 100 to 60% (Additional file 2: Fig. S1). The species distribution of the best matches (top-hit) against the non-redundant protein database indicated that the largest fraction of matching unigenes showed similarities with sequences of the copepod *Eurytemora affinis*, followed by the copepod *Acartia pacifica*, the cladocerans *Daphnia pulex* and *Daphnia magna* and the copepod *Pseudodiaptomus poplesia*. The other top-hit species were mainly crustaceans or arthropods, while three molluscs and one brachiopod were among the other first 20 top-hit species (Additional file 2: Fig. S1).

**Fig. 2:**
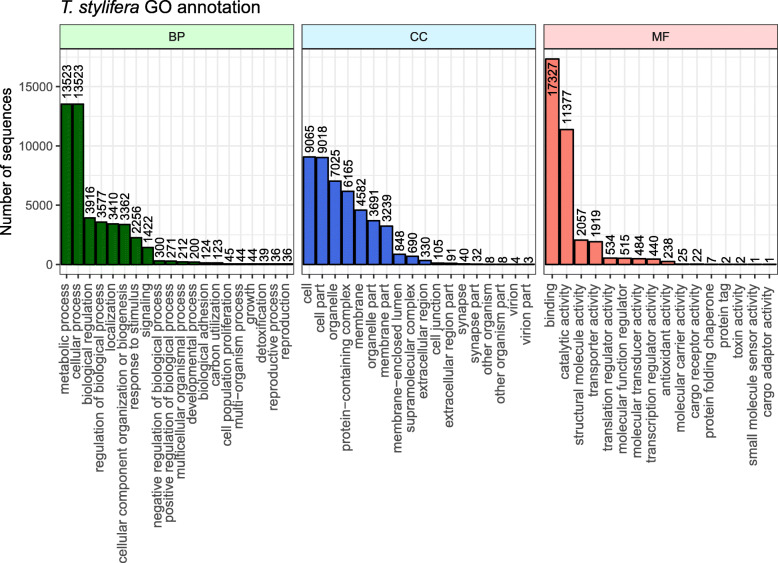
Blast2Go Gene Ontology (GO) annotation of *Temora stylifera* reference transcriptome (unigenes). The number of sequences assigned to the three GO classes Biological Process (BP), Molecular Function (MF) and Cellular Component (CC) are shown

Blast2Go annotation outputs showed that 31,346 unigenes, out of 62,648 that received significant matching in BLASTx, were functionally annotated (50.04%). In total, 126,358 GO annotation terms were assigned and distributed among GO categories for Biological Process (BP, 36.77%), Molecular Function (MF, 35.57%) and Cellular Component (CC, 27.66%) (Fig. 2). The majority of recognized unigenes were assigned to metabolic and cellular processes (29%), binding and catalytic activity (49.59 and 32.55%, respectively) and cell or cell part (both 20%).

### Differential expression analysis and transcriptome validation

Analysis of expression levels of *T. stylifera* unigenes between samples collected on May 30th and May 23rd showed that a total of 144 unigenes were differentially expressed (FDR *p* ≤ 0.05). In particular, on May 23rd 108 genes were down-regulated, while 36 genes were up-regulated. Of the total 144 differentially expressed sequences, 31 (6 up-regulated and 25 down-regulated) received GO assignment and functional annotation (Table 2).

**Table 2:** *Temora stylifera* differentially expressed unigenes

***Trinity ID number identifier***	***Length (bp)***	***log***_***2***_***-FC***	***p-adj***	***Description***	***GO IDs***
TRINITY_DN48953_c0_g1_i2	1109	−9.92	4.71^−06^	---NA---	
TRINITY_DN56306_c2_g1_i2	338	−7.09	0.0003	putative odorant-binding protein A5	
TRINITY_DN46479_c0_g2_i1	490	−6.06	0.02	TPA: hypothetical protein BOS_23229	
TRINITY_DN54322_c1_g1_i1	202	−5.18	0.01	OV-16 antigen-like	
TRINITY_DN56306_c3_g2_i2	262	−5.03	3.46^−17^	---NA---	
TRINITY_DN56306_c2_g2_i1	231	−4.78	1.69^−08^	putative odorant-binding protein A5	
TRINITY_DN56306_c0_g1_i1	279	−4.75	2.76^−16^	protein D3	
TRINITY_DN47252_c1_g3_i1	576	−4.60	0.01	collagen alpha-1(I) chain-like	
TRINITY_DN56306_c3_g1_i4	532	−4.50	1.30^−14^	putative odorant-binding protein A5	
TRINITY_DN44842_c0_g1_i2	513	−4.45	0.04	collagen alpha-1(I) chain-like	
TRINITY_DN59703_c3_g3_i1	328	−4.42	6.63^−10^	---NA---	
TRINITY_DN54322_c1_g2_i5	363	−4.29	0.001	protein D2	
TRINITY_DN40295_c1_g1_i1	287	−4.03	0.01	protein GVQW1-like	
TRINITY_DN57986_c1_g1_i2	211	−3.8	0.04	alpha-l1 nicotinic acetyl choline receptor	
TRINITY_DN51020_c2_g1_i1	774	− 3.76	0.03	---NA---	
TRINITY_DN47801_c76_g1_i1	255	−3.73	0.02	---NA---	
TRINITY_DN50358_c0_g1_i2	1964	−3.71	0.001	serine/threonine protein phosphatase Ppa2	F:GO:0016787
TRINITY_DN50277_c3_g1_i11	635	−3.51	0.003	hypothetical protein	
TRINITY_DN48256_c0_g1_i2	655	−3.39	0.02	cell wall-associated hydrolase	
TRINITY_DN47115_c1_g1_i13	430	−3.32	0.03	cell wall-associated hydrolase	F:GO:0016787
TRINITY_DN56650_c0_g2_i1	2126	−3.29	2.83^−08^	uncharacterized protein LOC111708691	
TRINITY_DN48918_c10_g1_i1	214	−3.13	0.0009	putative odorant-binding protein A5	
TRINITY_DN42279_c0_g1_i1	359	−3.03	2.61^−05^	collagen-like protein	
TRINITY_DN41737_c0_g1_i1	478	−2.95	0.01	---NA---	
TRINITY_DN42313_c0_g1_i4	230	−2.93	4.87^−14^	---NA---	
TRINITY_DN48948_c2_g1_i1	220	−2.85	3.64^−06^	putative odorant-binding protein A5	
TRINITY_DN48918_c8_g1_i1	223	−2.82	0.0006	39S ribosomal protein L38, mitochondrial	
TRINITY_DN59703_c2_g3_i1	286	−2.81	1.92^−12^	---NA---	
TRINITY_DN48067_c0_g1_i1	220	−2.81	3.34^−09^	uncharacterized protein LOC111712488 isoform X2	
TRINITY_DN59703_c2_g1_i2	335	−2.8	3.46^−17^	putative odorant-binding protein A5	
TRINITY_DN59703_c3_g1_i1	279	−2.79	1.82^−14^	---NA---	
TRINITY_DN50915_c1_g1_i13	1031	−2.78	0.04	IS1 transposase InsAB	
TRINITY_DN52417_c2_g3_i22	1136	−2.77	0.01	conserved hypothetical protein	
TRINITY_DN59703_c2_g2_i1	239	−2.68	6.39^−06^	protein D2-like	
TRINITY_DN48788_c0_g2_i4	1921	−2.68	0.009	dentin sialophosphoprotein isoform X2	
TRINITY_DN46462_c2_g1_i1	271	−2.6	6.82^−07^	---NA---	
TRINITY_DN48918_c6_g2_i2	368	−2.64	6.82^−07^	---NA---	
TRINITY_DN51686_c3_g3_i2	821	−2.63	0.01	---NA---	
TRINITY_DN46462_c2_g2_i3	229	−2.62	7.97^−05^	OV-16 antigen-like	
TRINITY_DN51574_c1_g2_i3	2092	−2.60	0.04	23S rRNA (guanosine(2251)-2′-O)-methyltransferase RlmB	
TRINITY_DN48918_c11_g1_i1	229	−2.55	0.01	OV-16 antigen-like	
TRINITY_DN52057_c0_g1_i2	1042	−2.54	0.0001	---NA---	
TRINITY_DN56306_c1_g1_i2	347	−2.53	8.91^−07^	OV-16 antigen-like	
TRINITY_DN48918_c5_g1_i2	287	−2.53	3.50^−07^	putative odorant-binding protein A5	
TRINITY_DN48918_c6_g1_i2	327	−2.53	6.98^−07^	OV-16 antigen-like	
TRINITY_DN48948_c0_g1_i1	202	−2.52	0.01	OV-16 antigen-like	
TRINITY_DN54592_c0_g1_i1	1761	−2.5	0.003	uncharacterized protein LOC111700481	
TRINITY_DN50660_c0_g2_i1	298	−2.44	0.03	---NA---	
TRINITY_DN43949_c0_g2_i2	213	−2.44	0.001	---NA---	
TRINITY_DN48948_c1_g2_i1	216	−2.39	0.0007	putative odorant-binding protein A5	
TRINITY_DN50660_c0_g1_i2	1041	−2.36	6.85^−06^	protein D3	
TRINITY_DN45228_c5_g1_i1	273	−2.36	2.47^−05^	---NA---	
TRINITY_DN53544_c0_g3_i2	1498	−2.34	0.01	altered inheritance of mitochondria protein 3-like	
TRINITY_DN48948_c1_g1_i17	315	−2.32	3.64^−06^	---NA---	
TRINITY_DN44278_c0_g1_i1	204	−2.3	0.03	cytochrome c oxidase subunit I (mitochondrion)	F:GO:0004129; C:GO:0005743; P:GO:0006123; P:GO:0009060; C:GO:0016021; F:GO:0020037; C:GO:0045277; F:GO:0046872; P:GO:1902600; P:GO:1902600
TRINITY_DN60327_c0_g1_i1	239	−2.22	0.02	---NA---	
TRINITY_DN38350_c0_g1_i1	250	−2.19	0.008	collagen alpha-1(XI) chain-like	F:GO:0005201; C:GO:0031012
TRINITY_DN60534_c0_g1_i3	490	−2.16	0.02	---NA---	
TRINITY_DN48918_c5_g2_i1	367	−2.13	3.37^−06^	protein D3	
TRINITY_DN48918_c10_g1_i1	214	−2.05	0.0009	sequestosome-1-like	
TRINITY_DN43949_c3_g1_i1	201	−2.04	0.01	---NA---	
TRINITY_DN46002_c0_g1_i1	370	−2.03	0.001	cAMP-responsive element-binding protein-like 2	F:GO:0003700; C:GO:0005667; P:GO:0006355; P:GO:0006355
TRINITY_DN55139_c4_g1_i3	305	−1.95	0.0006	---NA---	
TRINITY_DN53118_c0_g1_i2	1123	−1.95	0.0004	cytochrome b5-like	F:GO:0020037
TRINITY_DN46104_c2_g1_i22	715	−1.94	0.03	hypothetical protein T11_14937	
TRINITY_DN59998_c0_g2_i5	1026	−1.88	0.02	uncharacterized protein LOC111714070	
TRINITY_DN48367_c6_g8_i1	224	−1.86	0.04	malate dehydrogenase, mitochondrial	F:GO:0016491; P:GO:0055114
TRINITY_DN50562_c0_g1_i4	930	−1.86	0.002	cytochrome P450 2C9-like	F:GO:0005506; F:GO:0016705; F:GO:0020037; P:GO:0055114
TRINITY_DN55139_c4_g2_i1	254	−1.84	0.003	---NA---	
TRINITY_DN43949_c1_g1_i1	243	−1.83	0.0006	---NA---	
TRINITY_DN48564_c3_g1_i5	290	−1.79	0.02	---NA---	
TRINITY_DN61944_c3_g2_i1	569	−1.78	0.03	DNA ligase 1-like isoform X2	
TRINITY_DN58322_c1_g1_i3	335	−1.76	0.01	heat shock protein 70	
TRINITY_DN60002_c1_g1_i1	2425	−1.75	0.001	ariadne protein	
TRINITY_DN53340_c0_g1_i1	1921	−1.75	0.003	Leukocyte receptor cluster member 9	F:GO:0046872
TRINITY_DN55139_c4_g3_i1	236	−1.74	0.03	---NA---	
TRINITY_DN50562_c0_g2_i2	866	−1.74	0.002	cytochrome P450 CYP3034A1	F:GO:0005506; F:GO:0016705; F:GO:0020037; P:GO:0055114
TRINITY_DN58203_c2_g2_i3	1411	−1.7	0.03	---NA---	
TRINITY_DN44347_c0_g1_i1	2339	−1.7	7.72^−06^	Carboxylic ester hydrolase	
TRINITY_DN59831_c1_g3_i3	1224	−1.7	0.04	sterile alpha and TIR motif-containing protein 1 isoform X1	F:GO:0005515
TRINITY_DN51606_c3_g1_i1	201	−1.67	0.04	heat shock protein beta-1	
TRINITY_DN46142_c0_g1_i1	228	−1.62	9.03^−05^	peritrophins 3-A1 precursor	C:GO:0005576; P:GO:0006030; F:GO:0008061
TRINITY_DN49225_c2_g1_i3	356	−1.54	0.02	---NA---	
TRINITY_DN54543_c0_g1_i1	244	−1.54	0.007	peroxidase, putative	
TRINITY_DN45036_c0_g1_i4	818	−1.53	0.003	---NA---	
TRINITY_DN60327_c0_g3_i1	431	−1.53	0.02	e3 ubiquitin-protein ligase Mdm2-like isoform X1	
TRINITY_DN55139_c3_g1_i1	281	−1.52	0.005	---NA---	
TRINITY_DN61324_c6_g2_i2	221	−1.48	0.02	arylsulfatase B-like	
TRINITY_DN37862_c0_g1_i2	292	−1.45	0.04	arylsulfatase B-like	F:GO:0003824; P:GO:0008152
TRINITY_DN46682_c4_g1_i3	247	−1.4	0.01	ubiquitin	F:GO:0005515
TRINITY_DN55897_c0_g1_i1	2619	−1.37	0.007	sodium-dependent nutrient amino acid transporter 1-like	F:GO:0005328; P:GO:0006812; P:GO:0006836; C:GO:0016021
TRINITY_DN58435_c6_g2_i2	1086	−1.36	0.009	---NA---	
TRINITY_DN51606_c2_g1_i1	204	−1.32	0.03	heat shock protein beta-1-like	
TRINITY_DN57765_c0_g1_i1	850	−1.32	0.03	---NA---	
TRINITY_DN49317_c3_g1_i1	1068	−1.31	0.0008	Kelch-like protein 12	F:GO:0005515
TRINITY_DN51502_c2_g2_i2	599	−1.3	0.009	heat shock protein 70 B2	F:GO:0005524
TRINITY_DN57961_c5_g1_i1	370	−1.3	0.03	heat shock protein beta-1	
TRINITY_DN48866_c0_g1_i4	425	−1.29	0.03	uncharacterized protein LOC111717104	
TRINITY_DN61324_c6_g3_i3	552	−1.28	0.002	arylsulfatase B-like	F:GO:0003824; P:GO:0008152
TRINITY_DN61324_c6_g1_i1	1130	−1.28	0.003	arylsulfatase B-like	P:GO:0008152; F:GO:0008484
TRINITY_DN50806_c1_g2_i4	2085	−1.26	0.02	phosphatidylserine decarboxylase proenzyme, mitochondrial-like	F:GO:0004609; P:GO:0006544; P:GO:0006563; P:GO:0006566; P:GO:0008654; P:GO:0046486
TRINITY_DN46430_c2_g2_i2	627	−1.24	0.02	heat shock 70 kDa protein 1-like	F:GO:0005524
TRINITY_DN57961_c4_g1_i7	288	−1.19	0.03	heat shock protein beta-1	
TRINITY_DN51606_c1_g1_i7	760	−1.18	0.04	heat shock protein beta-1-like	
TRINITY_DN54808_c0_g1_i1	1258	−1.14	0.03	arginine kinase	F:GO:0016301
TRINITY_DN56814_c0_g1_i3	876	−1.13	0.004	arylsulfatase B-like	P:GO:0008152; F:GO:0008484
TRINITY_DN56639_c0_g1_i2	3248	−1.05	0.03	protein unc-45 homolog B	F:GO:0005515
TRINITY_DN59770_c0_g1_i1	2702	−0.96	0.01	solute carrier organic anion transporter family member 2A1	F:GO:0005215; F:GO:0005515; C:GO:0016020; P:GO:0055085
TRINITY_DN48929_c1_g2_i2	232	0.99	0.01	---NA---	
TRINITY_DN48585_c5_g2_i2	267	1.01	0.0008	---NA---	
TRINITY_DN50261_c1_g1_i4	259	1.07	0.01	---NA---	
TRINITY_DN46130_c0_g2_i2	257	1.07	0.01	transforming growth factor-beta-induced protein ig-h3-like	
TRINITY_DN58926_c0_g1_i1	2210	1.11	0.005	organic cation transporter protein-like	C:GO:0016021; F:GO:0022857; P:GO:0055085
TRINITY_DN47352_c0_g1_i11	566	1.11	0.002	uncharacterized protein LOC111697309	F:GO:0005506
TRINITY_DN44753_c2_g1_i8	282	1.13	0.003	---NA---	
TRINITY_DN46080_c1_g2_i1	298	1.23	0.003	---NA---	
TRINITY_DN47755_c1_g1_i2	231	1.34	0.001	---NA---	
TRINITY_DN39167_c0_g1_i1	458	1.43	0.03	vitellogenin receptor	
TRINITY_DN49031_c4_g1_i1	214	1.44	0.002	---NA---	
TRINITY_DN56235_c0_g1_i2	867	1.5	0.002	facilitated trehalose transporter Tret1-like	C:GO:0016021; F:GO:0022857; P:GO:0055085
TRINITY_DN48929_c1_g1_i1	272	1.57	0.0004	---NA---	
TRINITY_DN53823_c0_g4_i3	1704	1.58	0.01	Facilitated trehalose transporter Tret1	C:GO:0016021; F:GO:0022857; P:GO:0055085
TRINITY_DN50724_c4_g1_i1	259	1.68	0.003	---NA---	
TRINITY_DN47273_c4_g1_i1	201	1.77	0.002	---NA---	
TRINITY_DN47638_c5_g1_i1	269	2.09	0.004	---NA---	
TRINITY_DN46792_c0_g1_i13	419	2.15	0.001	---NA---	
TRINITY_DN48936_c0_g1_i1	2363	2.16	0.003	uncharacterized protein LOC111698428	
TRINITY_DN46832_c5_g1_i3	221	2.24	0.04	---NA---	
TRINITY_DN46134_c9_g2_i1	368	2.34	0.01	---NA---	
TRINITY_DN44649_c1_g3_i4	295	2.42	6.52^−10^	---NA---	
TRINITY_DN48983_c0_g1_i1	2068	2.61	0.03	uncharacterized protein LOC111696662	
TRINITY_DN47725_c0_g1_i1	324	2.75	0.01	---NA---	
TRINITY_DN57759_c4_g3_i1	224	3.11	2.36e-07	---NA---	
TRINITY_DN43876_c0_g1_i1	848	3.42	1.39^−06^	---NA---	
TRINITY_DN26125_c0_g1_i1	1469	3.55	0.02	probable serine/threonine-protein kinase samkA	
TRINITY_DN47135_c3_g1_i7	240	3.57	3.94^−08^	---NA---	
TRINITY_DN41595_c0_g1_i1	1708	3.63	0.02	uncharacterized protein LOC111704026 isoform X2	
TRINITY_DN44116_c1_g1_i1	1536	3.76	0.0003	neuronal acetylcholine receptor subunit alpha-10-like isoform X1	F:GO:0004888; F:GO:0005230; P:GO:0007165; C:GO:0016021; P:GO:0034220
TRINITY_DN54042_c0_g1_i1	2084	4.07	0.01	---NA---	
TRINITY_DN57931_c1_g6_i1	814	5.11	0.01	N-acylglucosamine 2-epimerase	
TRINITY_DN40267_c0_g1_i2	261	5.41	0.003	putative ATP-dependent RNA helicase me31b	F:GO:0003676; F:GO:0005524
TRINITY_DN52242_c0_g2_i2	1016	6.73	1.32^−09^	---NA---	
TRINITY_DN52242_c0_g1_i3	312	7.13	0.0003	---NA---	
TRINITY_DN49250_c2_g3_i1	201	8.75	0.01	---NA---	

Trinity ID number with predicted gene and isoform identifiers, length (bp), log_2_-Fold-Change (log_2_-FC), adjusted *p*-value (*p*-adj) of statistical analysis (FDR) for each predicted genes, sequence description and functional annotation as provided by Blast2Go for the longest isoform. Unigenes are listed from the most down-regulated to the most up-regulated one, as indicated by log_2_-FC

**Fig. 3:**
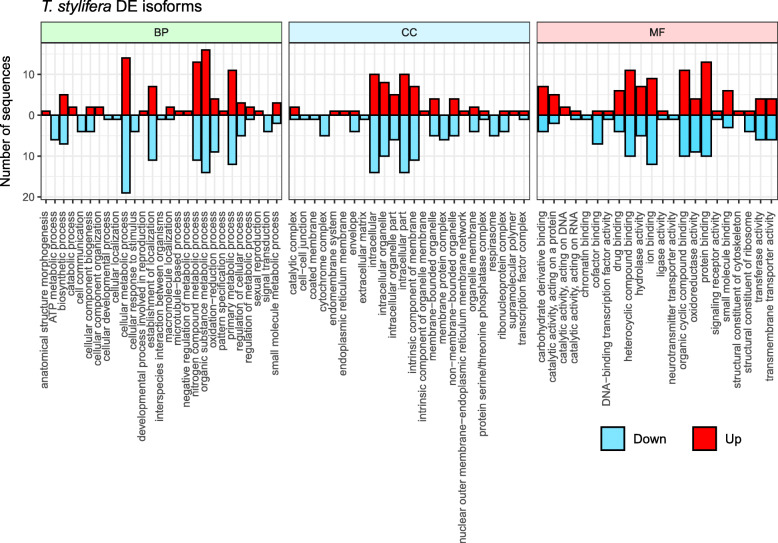
Blast2Go Gene Ontology (GO) annotation of the differentially expressed transcript isoforms in *Temora stylifera*. First 27 terms of each category:Biological Process (BP), Cellular Component (CC) and Molecular Function (MF) are shown along the x-axis; the number of sequences assigned to each GO term within each GO category is displayed on the y-axis. Down-regulated sequences are indicated by blue bars and up-regulated sequences by red bars

In order to have a wider spectrum of gene functions and to allow a more detailed description of the molecular responses of *T. stylifera* females on the two sampling dates, differential expression analysis was performed also on transcript isoforms. Among isoforms, 331 sequences were differentially expressed (FDR *p* ≤ 0.05), 199 were down-regulated and 132 were up-regulated. In total, 119 differentially expressed isoforms received GO assignment and were functionally annotated (58 up- and 61 down-regulated) (Additional file 3: Table S2). In total, 563 GO terms were associated to the differentially expressed isoforms and were assigned to the three main GO categories, which were almost equally divided among BP (37.18%) and MF (34.66%), while a smaller fraction described the CC category (28.16%). Analysis of GO distribution among the three main categories was also repeated dividing up- and down-regulated isoforms (Fig. 3). Results showed similar number of up-regulated and down-regulated sequences in the different GO in terms of BP, MF and CC categories.

Interestingly, a number of specific GO terms contained isoforms that were exclusively up- or down-regulated on May 23rd in comparison to May 30th (Fig. 3). Among BP sub-categories, sequences involved in ATP metabolism (6 sequences), cell communication (4 sequences), cellular response to stimulus (4 sequences), signal transduction (4 sequences), cellular development (1 sequence), cellular localization (1 sequence) and organism interaction (1 sequence) were specifically down-regulated. In contrast, sequences involved in catabolic processes (2 sequences), cellular component organization (2 sequences), anatomical structure morphogenesis (1 sequence), microtubule-based process (1 sequence), negative regulation of metabolic process (1 sequence), pattern specification process (1 sequence) and sexual reproduction (1 sequence) were exclusively up-regulated.

**Fig. 4:**
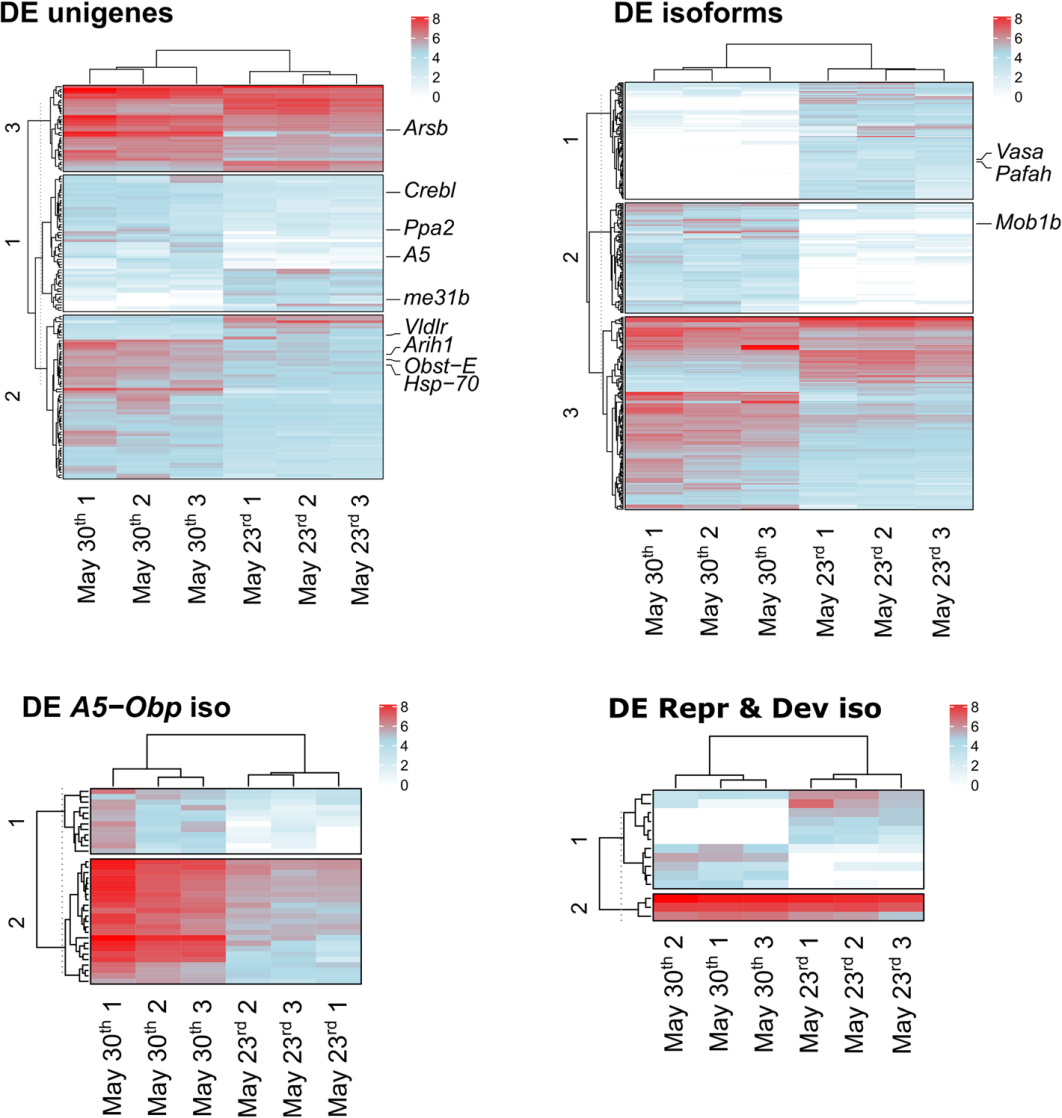
Heat-maps of differentially expressed sequences expressed as log-transformed raw counts. Euclidean distance was considered as distance measure and variables were clustered on the basis of the complete-linkage method. Numbers near the dendrogram of variables (R-mode) describe the main clusters identified for sequences. Cluster position of the selected differentially expressed unigenes and isoforms used for transcriptome validation is indicated. Dendrograms on the top of the heat-maps describe grouping of samples on the basis of complete-linkage clustering method (Q-mode). Data refer to the three replicates of wild *Temora stylifera* collected on the 30th of May 2017 and the 23rd of May. Heat-maps at the top show raw counts of differentially expressed unigenes (DE unigenes) and isoforms (DE isoforms). Heat-maps at the bottom display log-transformed raw counts of *A5 Odorant binding proteins* (DE *A5-Obp* iso) isoforms and isoforms annotated as sequences involved in reproduction and development (DE Repr & Dev iso)

Consistent differences between replicates collected on May 30th and May 23rd were supported by clustering among objects (Q-mode analysis) described by raw counts of both the differentially expressed unigenes and isoforms (Fig. 4). Also, such clustering was confirmed when raw counts of isoforms involved in specific molecular pathways were selected and analysed separately (Fig. 4).

Most of the significantly down-regulated unigenes and isoforms on May 23rd were described as *A5 Putative Odorant Binding Protein* (*Obp*, annotated as sequences involved in ‘response to stimulus’). Other down-regulated unigenes were annotated as sequences related to developmental metabolic processes (involving chitin and collagen), protein ubiquitination, response to stress (mainly *Heat Shock Protein 70*), oxidation-reduction reactions and hydrolase activities. Similarly, additional down-regulated isoforms were involved in respiration, protein binding, transmembrane transport and cellular development. The significantly up-regulated unigenes and isoforms were mainly involved in reproduction, cell development and proliferation (e.g. *Vitellogenin-like unigenes*, *RNA Helicase* and *Lipoprotein Receptor* unigenes), transmembrane transport and reception activity.

Based on these results, 9 unigenes and 3 isoforms were selected as Gene of Interests (GOIs) for transcriptome validation through RT-qPCR analysis depending on function, fold-change, significance (adjusted *p*-values), sequence length, E-value and sequence similarity percentage. Although unigenes offered a narrower array of functions in comparison to isoforms, most primers were selected from unigenes to reduce redundancy due to multiple transcript isoforms within the same Trinity gene cluster. Amplicons were all in the range of 111–228 bp and showed primer amplification efficiencies between 1.9 and 2.1. The full list of primer sequences for these selected sequences of interest is shown in Table 3.

**Table 3:** List of unigenes and isoforms tested for *Temora stylifera* transcriptome validation

Name	A_**L**_ (bp)	log_**2**_-FC	***p***-adj		Primers	%E
*A5*^1^	130	−4.61	9^−12^	F	GCCTGTTGCCGGAAACTTTT	110
				R	TTCTGGGCCGTCATTGACTC	
*CREBL*^1^	111	−2.04	2^−3^	F	GTACAAGCTGGAGAGGAGTCG	103
				R	GCCTTATTTGCCCTCTCCCT	
*me31b*^1^	136	5.42	4^−3^	F	TTCTGGACGAAGCGGACAAG	114
				R	CGCATGAAGGACTCGACTGT	
*Ppa2*^1^	140	−3.72	1^−3^	F	GCTTTGCCTTAAACTGCGCT	113
				R	CGGCAGGTAGTCAAACAGGT	
*Obst-E*^1^	228	−1.62	9^−5^	F	CAAGATCGACTGTCTGGGCA	103
				R	CGAGCCTTTCCACTCCACTT	
*ARIH1*^1^	200	−1.76	2^−3^	F	AGATGTGGGGCTGCAACTAC	106
				R	CTCAATCTTCTCCAGCGGCA	
*ARSB*^1^	116	−1.29	4^−3^	F	AACAACAGGGGCTTCAACCA	108
				R	TCAAACTCTGGCACCCTGTC	
*Hsp70*^1^	133	−1.25	3^−2^	F	CCATTCAGGTCTACGAGGGC	102
				R	TTGGCGTCAATGTCGAAGGT	
*VLDL1*^2^	132	1.44	4^−2^	F	ATCGCAGGGTCATTGTCCAG	112
				R	TGCGTATGTCTCGACCAGTG	
*MOB1B*^2^	156	−9.48	4^−10^	F	TTGTCCTGTCATGTCGGCAG	89
				R	TTGCTGGGGAACAAGGACTC	
*Vasa*^2^	138	7.14	3^− 4^	F	CGCCTTCAACGATCTCCAGT	103
				R	GCCGAGAACATAAGGGTGGT	
*PAFAH*^2^	163	6.93	2^−3^	F	GCCTTCACCTCGCTCTTCAG	87
				R	AGGCGTATCGATTGCAACCT	

Name, amplicon length (A_L_) (bp), log_2_-Fold-Change (log_2_-FC), adjusted *p*-value (*p*-adj), primer sequence and amplification efficiency percentage (%E) are shown. ‘^1^’: unigenes; ‘^2^’: isoforms

For RT-qPCR analysis, the expression of GOIs was normalized considering 18S ribosomal RNA (*18S*) and Ubiquitin (*Ubi*) as reference genes (Additional file 4: Table S3). These two genes were indicated as the most stable ones among the five candidates selected as potential reference sequences according to results provided by RefFinder [60] (mean of ranking values: 1 and 1.68, respectively).

**Fig. 5:**
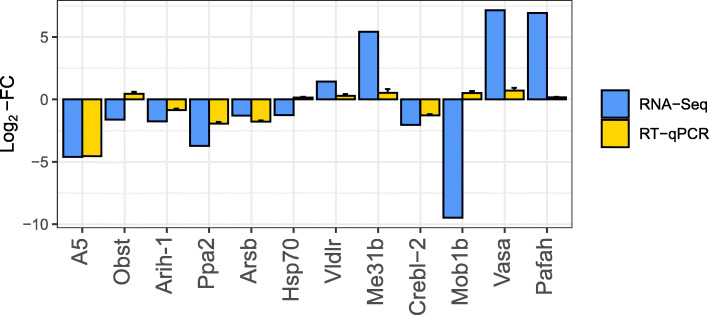
Comparison between RNA-Seq and RT-qPCR in *Temora stylifera*. Relative expression ratio (log_2_-Fold-Change, FC) of 9 unigenes (*A5*, *Obst*, *Arih1*, *Ppa2*, *Arsb*, *Hsp-70*, *Vldlr*, *Me31b* and *Crebl2*) and 3 isoforms (*Mob1b*, *Vasa* and *Pafah*) in *Temora stylifera* from May 23rd vs 30th samples, measured through RNA-Seq (blue bars) or RT-qPCR (yellow bars). For RT-qPCR results, bars represented mean ± SD values and data are normalized to RNA *18S* and *Ubi* reference genes

Normalized expression of the selected GOIs for transcriptome validation supported RNA-Seq results, because 9 sequences out of 12 reflected the same up- or down-regulation patterns as in the differential expression analysis (Fig. 5). However, the genes *Protein Obstructor E* (*Obst*) and *Heat-Shock Protein 70* (*Hsp70*) as well as the isoform *MOB Kinase Activator 1B* (*Mob1b*) showed an opposite trend in comparison to RNA-Seq results. In general, log_2_-fold change indicating differential expression in *T. stylifera* females collected on May 23rd in comparison to those collected on May 30th was larger in the RNA-Seq output than RT-qPCR results. In particular, RNA-Seq log_2_-fold-changes of the three selected isoforms (*Mob1b*, *Vasa* and *Pafah*; log_2_-FC = − 9.48, 7.14 and 6.93, respectively) were much higher than RT-qPCR values. On the contrary, the expression ratio obtained after RT-qPCR analysis for the unigenes *A5 Obp* (log_2_-FC = − 4.6), *Arih1* (log_2_-FC = − 0.85), *Ppa2* (log_2_-FC = − 1.94), *Arsb* (log_2_-FC = − 1.78) and *Crebl* (log_2_-FC = − 1.28) resembled results obtained from the RNA-Seq analysis (log_2_-FC = − 4.55, − 1.76, − 3.72, − 1.29 and − 2.03, respectively).

## Discussion

Estimating early-life history variables such as egg production, hatching success and survival rates of non-feeding (NI/NII) nauplii in field and laboratory studies has been traditionally considered to infer recruitment potential in copepod populations [18, 34, 40, 42]. In this perspective, understanding which molecular mechanisms contribute to define naupliar viability may help predict reproductive responses of natural copepod populations. In particular, our DE analysis may allow elucidating molecular mechanisms affecting naupliar viability in *T. stylifera* females collected from natural populations in the Gulf of Naples (GoN). Information about environmental variables, phytoplankton community composition and abundance, oxylipin-per-litre concentrations and oxylipin-per-diatom-cell production can elucidate the influence of these variables on molecular responses of copepod females and final naupliar viability. While on a side environmental variables were proposed as major factors affecting *T. stylifera* population in the GoN [44, 48], on the other side wide evidence has been provided about the detrimental effect of phytoplankton-derived oxylipins on the reproductive success of grazer copepods [9, 17–22, 24, 36], possibly in relation to differential expression of key genes involved in reproduction and stress responses [3, 26–33, 35].

The present transcriptome analysis resulted in 268,665 transcripts (isoforms) and 120,749 predicted genes (unigenes). These numbers were sensibly higher than results reported in other calanoid copepods, such as *Calanus helgolandicus* [33], *C. finmarchicus* [61], *Calanus sinicus* [62], *Temora longicornis* [57] and *Acartia tonsa* [58]. Rather, our de novo transcriptome assembly for *T. stylifera* was much closer to results presented by [52] for *C. finmarchicus*, because these authors reported 241,140 transcripts and 124,157 components (corresponding to our Trinity predicted genes). The vast majority of the 20 top-hits BLAST species for the present transcriptome was represented by crustaceans and the first two top-hit species were the copepods *Eurytemora affinis* and *Acartia pacifica*. High affinity of the blasted copepod sequences to crustaceans and arthropods is increasing in the last years [33, 57, 63] and this indicates that publicly available copepod genomic resources are improving. Nonetheless, only nearly 50% of *T. stylifera* transcripts and predicted genes found hits in BLASTx and around 25% of these sequences received GO annotation. These low percentages demonstrate that much effort is still needed to improve genomic resources for copepods, even if the present results suggest improvements from previous de novo transcriptomic analyses [61, 62]. Annotation output of *T. stylifera* transcriptome showed that most unigenes were annotated in metabolic and cellular processes BP sub-categories and in cell or cell part CC sub-categories, in good agreement with previous transcriptomic studies on calanoid copepods [33, 49, 57, 58, 62].

After an overview of the de novo transcriptomic assembly and annotation, we inspected if our differential analysis could help infer maternally-related molecular processes involved in naupliar survival. We compared expression rates in *T. stylifera* adult females generating highly viable nauplii (on May 30th) with those of females generating nauplii with low survival rates (on May 23rd) and found that differentially expressed sequences were mostly involved in signal perception and transduction as well as in reproductive and developmental processes.

More in particular, most down-regulated transcripts on May 23rd were *Putative Odorant-Binding Proteins*. Most of them were *A5-Binding Proteins* (belonging to the Phosphatidylethanolamine-Binding Protein class), whose expression has been found in the palps and antenna of the mosquito *Anopheles gambiae* [64]. *Odorant binding proteins* (*Obps*) are part of the arthropod olfactory system, which has been extensively characterized in the model fly *Drosophila melanogaster* [65]. In this organism, suppression of *Obp* expression mediated an altered behavioural response [66] and modulation in the ingestion of bitter tastants [67]. In particular, inhibition of *Obp A5* in *D. melanogaster* females led to differential responses to benzaldehyde and citral, to increased ingestion of N-phenylthiourea and papaverine as well as to reduced consumption of caffeine and denatonium benzoate. These results support that *Obps* in *Drosophila* are involved in detection of harmful chemicals. In fact, *Obps* were found to mediate a negative response (i.e. avoidance) of *Drosophila sechiella* to phyco-toxins, because the *Obps 57d* and *57e* allowed recognition of the plant-derived hexanoic acid and octanoic acid [68, 69]. One of the most important features highlighted in the *Obp* modulation as response to olfactory stimuli in *D. melanogaster* is that *Obp* gene expression is combinatorial [66, 67]. This means that the response to an odorant stimulus is regulated by the expression of multiple *Obps*. Copepods base their feeding behaviour on mechanosensory setae, chemosensory sensilla (i.e. aesthetascs) or bimodal sensilla, present on antennules A1, antennas and the maxilliped. Such sensory organs allow copepods to distinguish prey morphology, to detect foraging stimuli and avoid food items on the basis of chemicals [70]. In spite of combinatorial regulation as in *D. melanogaster*, our differential analysis revealed down-regulation of only *A5 Obps* along with those sequences described as *OV-16*. This result could suggest specific involvement of *A5 Obp* in the response to algal-derived toxic compounds in *T. stylifera* females. Despite major differences in signal perception between aquatic and non-aquatic environments [71, 72], *Obps* in arthropods likely encode for proteins displaying similar functions. Several experiments have reported different copepod behaviours in response to specific preys or compound classes [73–75]. In particular, dissolved polyunsaturated aldehydes (PUAs), a class of volatile oxilipins, induced attraction of *T. stylifera* females in odour-choice experiments, suggesting that the copepods showed a behavioural preference for oxylipins [73]. Total oxylipin-per-litre concentration and oxylipin-per-diatom-cell production were both higher on May 23rd than 30th. Down-regulation of *A5 Obps* in *T. stylifera* could potentially suggest that detection capacity of phytoplankton-derived harmful molecules was reduced in copepod females collected on May 23rd, leading to unselective feeding, higher ingestion of oxylipin producing diatoms and negative effects on larval survival.

In contrast to wide functional information of *Obp* regulation in *D. melanogaster*, to the best of our knowledge this is the first time that differential expression of these sequences is reported in a copepod. Down-regulation of *A5 Obp* in *T. stylifera* females was confirmed by RT-qPCR results (log_2_-FC in RNA-Seq = − 4.6; log_2_-FC in RT-qPCR = − 4.55) and could have played a relevant role in determining ingestion of harmful preys and low reproductive output on May 23rd. These results suggest that *Obps* can represent important target genes and proteins to investigate how copepods respond to phytoplankton-derived chemicals.

In addition to sequences involved in signal perception, differential analysis supported altered functions in signal transduction, because four sequences classified in the signal transduction BP sub-category were down-regulated. The *1-Phosphatidylinositol 3-Phosphate 5-Kinase* (*Fab1b*) showed the strongest down-regulation (log_2_-FC of − 10.34). This enzyme catalyses phosphorylation of the Phosphatidylinositol 3-Phosphate to synthesize Phosphatidylinositol 3,5-Bisphosphate, PI (3,5) P2. This molecule is one of the seven regulatory Polyphosphoinositides (PPIn) that are ubiquitous in eukaryotes, where they regulate membrane trafficking in endosomes and lysosomes [76]. Ablation of the Phosphoinositide kinase *Pikfyve* (the FAB1B counterpart in mammals) and consequent depletions in intracellular levels of PI (3,5) P2 have been demonstrated to induce embryonic and neonatal lethality in mammals [77]. In fact, PIP2 acts precursor for Phosphatidylinositol triphosphate (PIP3), a membrane lipid acting as a docking site for proteins involved in cell survival, proliferation and differentiation [78].

In support to the down-regulation of *Fab1b*, the significant up-regulation of the *Platelet-Activating Factor Acetyl Hydrolase* (*Pafah*) suggests repression of the PPIn pathway. In fact, PAFAH is a particular type of Phospholipase A2 that deacetylates the Platelet-Activating Factor (PAF), inducing loss in its activity [79, 80]. PAF is one of the most potent lipid mediators and occurs in membrane phospholipids. In mammals, PAF is supposed to regulate reproductive cycle and pregnancy [80] by initiating the enzymatic reactions leading to activation of PIP3, which determines high survival of offspring [78]. PIP3 also induces calcium transients that activate the cAMP-responsive element-binding protein (CREB) through phosphorylation. CREB is known to be a fundamental regulator of the signalling mechanisms in the cell and is fundamental during embryonic development [78]. In line with down-regulation of *Fab1b* and up-regulation of *Pafah*, *Crebl2* (*cAMP-Responsive Element-Binding Protein-Like 2*) was down-regulated in copepod females collected on May 23rd, as also validated by RT-qPCR results. Therefore, differential expression of this gene could have key implications for cell cycle and cell differentiation [78, 81]. Additionally, PAFAH can target phospholipids and participate to oxidative-stress responses as well as to regulation of fertility and apoptosis [82]. In humans, the glycosylphosphatidylinosotol (GPI) is known to induce pro-inflammatory responses [83, 84], thus differential expression of *Fab1b* and *Pafah* could potentially suggest also dysregulation of the inflammatory response as a consequence of an oxidative stress potentially mediated by oxylipins. Also in this perspective, a synergic response of PAFAH, FAB1B and CREBL in *T. stylifera* females could have contributed to determine low naupliar survival on the 23rd of May.

Low naupliar survival rates could also relate to down-regulation (validated also by RT-qPCR) of the *Serine/Threonine Protein Phosphatase 2A* (*Ppa2*), because the important role of this enzyme in regulation of apoptosis has been documented [85]. Serine/Threonine phosphatases, such as PP1, PP2A and PP2B, mediate de-phosphorylation of Bcl-2/Bcl-X-associated death promoter (BAD), a pro-apoptotic protein, thus leading to cell death. PP2A activity can be regulated by Phosphodiesterases (PDEs), which repress PP2A activity through regulation of cAMP levels [86], influencing oocyte maturation in pre-ovulatory follicles and meiosis in oocytes [87, 88]. As highlighted by our DE analysis, *Pde* was down-regulated and this response could have led to higher cAMP levels and stronger stimulation of *Ppa2* as compensatory mechanism to re-equilibrate dysregulated apoptosis.

Differentially expressed sequences directly involved in reproduction and development processes may also explain strong differences in naupliar survival rates at the molecular level. The enzyme *Methylenetetrahydrofolate Dehydrogenase* (*Mthfd*) was listed among the up-regulated transcripts (log_2_-FC of 4.45). Folate one-carbon metabolism plays a fundamental role in development and disease response of animals [89, 90]. The folate metabolism is complex, because it involves the activity of several enzymes. The active folate form is the tetrahydrofolate (THF) [91], which is a cofactor involved in the biosynthesis of thymidylate, purines, glycine, serine and homocysteine [92]. The enzyme methylenetetrahydrofolate dehydrogenase (MTHFD) converts tetrahydrofolate (THF) into 10-Formyl, 5,10-Methenyl and 5,10-Methylene derivatives which are key cofactors in the de novo synthesis of DNA [93]. For example, altered folate expression has been shown to drastically reduce fecundity of *D. melanogaster* females and to induce abnormal development in offspring [90]. Also, dys-regulation of the gene expression pathway associated to folate metabolism was recently reported in *Calanus helgolandicus* females feeding on the oxylipin-producing diatom *Skeletonema marinoi* [33].

In this perspective, altered expression of *Mthfd* could potentially contribute to explain impairment of the final reproductive success of *T. stylifera* in our survey.

Also, the *Trinucleotide repeat-containing gene 18* (*Tnrc18*) was significantly down-regulated. This protein is involved in cell differentiation and its contribution to development of copepod larvae may be important, because this gene is known to have a chromatin binding activity and has been proposed as a new marker gene for development in zebrafish embryos [94]. Additionally, *Dermatopontin* (*Dpt*) was also down-regulated. This gene encodes for a protein involved in collagen fibril orientation [95]; therefore its down-regulation and consequent impaired function could have led to negative effects on correct tissue formation in *T. stylifera* nauplii [96].

Previous laboratory experiments have reported variations in the expression of *α-* and *β-Tubulin* in copepods fed oxylipin-producing diatoms [3] and similar responses were quantified from natural populations of the copepod *C. helgolandicus* [35]. β-TUBULIN affects spindle formation and cell division; the up-regulation of this transcript could suggest an active response of *T. stylifera* females to high oxylipin concentrations potentially impairing reproductive success. In line with *β-Tubulin*, also the *Protein Maelstrom* (*Mael*) was up-regulated. MAEL has been reported as a required factor for the correct positioning of the microtubule-organizing centre (MTOC), in *D. melanogaster* [97]. Moreover, not only does MAEL act as a repressor for micro-RNA-7 in the nucleus, guaranteeing proper differentiation of germline cells [98], but also contributes to oocyte determination [99].

Other sequences relevant for cell development and reproduction were up-regulated in *T. stylifera* collected on May 23rd, such as *me31b* (*ATP-Dependent RNA Helicase*), *Vldlr* (*Very Low-Density Lipoprotein Receptor*) and *Vasa* (*ATP-Dependent RNA Helicase Vasa*). These genes are known to play key roles in germ cell formation [100, 101] as well as in oocyte development and differentiation [102–104].

Down-regulation was observed also for other sequences encoding for key functions in development and reproduction. Among them, *Obst-E* (*Protein Obstructor E*) is known to encode for a chitin-binding protein [105] and is crucial for the correct cuticle development in arthropods [106]. In line with down-regulation of *Obst-E*, the unigenes *Kelk 12* (*Klhl12*) and *Arih1* (*E3 Ubiquitin Protein Ligase*, validated through RT-qPCR) were also down-regulated. *Klhl12* mediates ubiquitination and regulates the Wnt signalling pathway, thus playing a key role in collagen export and embryonic cell development [107]. Similarly, *Arih1* codifies for an E3 Ubiquitin-protein ligase and, together with *Obst-E*, was included by [51] in the list of the 31 genes involved in reproduction, growth and development in the calanoid copepod *Eurytemora affinis*, where ubiquitin genes were reported to affect gametogenesis. The down-regulated transcript *Hsp-70* (*Heat-Shock Protein 70*) has close affinity with E3 Ubiquitin-protein ligase and it has been demonstrated to have a critical role in preventing apoptosis [108]. *Hsp-70* expression is also involved in thermo-tolerance mechanisms as well as in protection to xenobiotic exposure and to general stress in copepods [29, 109–111]. Previous laboratory studies have reported down-regulation of *Hsp-70* in the copepod *C. helgolandiscus* fed oxylipin-producing diatoms [26, 28, 112], suggesting that down-regulation of *Hsp-70* in *T. stylifera* could also have occurred in response to these chemicals.

Differential analysis also suggested altered functions in protein endocytosis and polysaccharide degradation, which we hypothesize could have contributed to the low naupliar viability observed on May 23rd. Among these sequences, the *Adaptor Protein Complex 2* (*Ap-2*) showed a significant down-regulation (log_2_-FC of − 7.41). *Ap-2* is involved in clathrin-dependent endocytosis in which proteins are incorporated into vesicles surrounded by clathrin (CVV) [113]. *Ap-2* is therefore strictly related to both the endosomal and the lysosomal systems [114] and is essential to fundamental cellular processes [113]. Also, *Arsb* (*Arylsulfatase B*), mainly related to digestion of polysaccharides [115], was a an additional down-regulated unigene and such differential expression was supported by RT-qPCR results. ARSB plays a central role in degradation of glycosaminoglycans (GAG) and altered regulations of this gene can result in lysosomal excretion of polysaccharides [116].

In final instance, several differentially expressed transcripts involved in the oxidative phosphorylation chain, such as *Cytochrome oxidase subunit I* and *Cytochrome oxidase subunit II,* were also down-regulated (log_2_-FC ranging from − 2.73 to − 5.75). Considering that oocyte mitochondrial dysfunction in terms of respiration and electron transport can alter fertility and embryo development [117, 118], this response is in line with the low naupliar viability observed in samples collected on May 23rd. The potential effects of phytoplankton-derived oxylipins on these transcripts is plausible, because significant down-regulation of the *Cytochrome P450–4* (*Cyp*) was also reported in wild *C. helgolandicus* females from the Northern Adriatic Sea, when collected at sampling sites showing high diatom and oxylipin concentrations [35]. These results were confirmed in the laboratory after feeding *C. helgolandicus* females from the Northern Adriatic Sea and the English Channel with the oxylipin-producing diatom *Skeletonema marinoi* [26].

## Conclusions

In general, information collected at the transcriptomic level revealed that *T. stylifera* females generating nauplii with low viability showed down- or up-regulation of sequences encoding for proteins involved in metabolic processes crucial for final reproductive success. In particular, key genes involved in ATP synthesis, cell differentiation and signal transduction could explain at a molecular level the maternally-mediated low naupliar survival observed on May 23rd. Differentially expressed sequences can also indicate molecular pathways regulated by *T. stylifera* females to counteract negative reproductive potential. In this perspective, some differentially expressed genes are known to encode for interconnected proteins participating in cellular biochemical patterns finally regulating apoptosis, cell cycle, yolk protein precursors (YPP), gene expression and autophagy. These processes could partially disentangle coordinated molecular pathways leading to altered naupliar viability. Interestingly, key genes involved in detoxification such as aldehyde dehydrogenases, superoxide dismutase or glutathione synthase were not differentially expressed in *T. stylifera* from the GoN.

Although we cannot exclude that biotic and abiotic factors not considered in this study may have mediated molecular responses of copepods, our data suggest that temperature, oxygen, pH and salinity played a marginal role, because these factors remained constant in the two sampling dates. Also, *T. stylifera* responses did not seem to be mediated by food deficiency, because differences in faecal pellet production were not significant despite the higher phytoplankton concentration on the 30th of May than the 23rd. Interestingly, both oxylipin-per lire concentrations and oxylipin-per-diatom-cell production were higher on the 23rd of May. Involvement of these chemicals in differential molecular responses and impaired naupliar survival in copepods from natural populations is plausible. Down-regulation of *A5 Obp* could suggest impaired detection of harmful preys in females collected on May 23rd, higher ingestion of oxylipins and lower naupliar survival. Indeed, our interpretation is tempting, but needs to be supported by larger datasets exploring wider temporal variations in the reproductive success and the molecular responses of natural *T. stylifera* populations. Our transcriptomic analysis could offer a guideline to identify biomarker genes of interest explaining at a molecular level copepod reproductive responses to biotic and abiotic variables, both in laboratory experiments and in the natural environment.

## Methods

### *Field sampling and physiological responses of Temora stylifera*

Environmental variables (temperature, salinity, pH and oxgen), phytoplankton community composition and phytoplankton-derived oxylipins were measured from surface water sampled at the Long-Term Ecological Research station-MareChiara (LTER-MC) [119] on the 23rd and the 30th of May 2017 as described by Russo et al. [47]. Briefly, abiotic variables were measured by deploying a SBE911 CTD. For phytoplankton and oxylipin analysis, surface water was collected with a bucket deployed from the boat. Phytoplankton cells were left to sediment in a Utermöhl chamber and counted under a Zeiss Axiovert200 optic microscope (Zeiss, Oberkochen, Germany). For oxylipin analysis, phytoplankton cells were accumulated on polycarbonate filters (2 μm mesh size). Cells were re-suspended with water and sonicated. Oxylipins were finally extracted with dichloromethane and methylated before the LC-MS/MS analysis, performed with a Q Exactive Hybrid Quadrupole Orbitrap (Thermo Scientific, Waltham, USA) using a methanol:water gradient [14].

Zooplankton samples were collected the same days at LTER-MC by the SZN Unit through oblique towing of a 200 μm Nansen net (113 cm mouth diameter) equipped with a 200 μm filtering cod-end, on-board of the R/V “Vettoria”. A maximum of 15 females of *T. stylifera* were sorted from the zooplankton community. They were individually incubated (20 °C, 12 h,12 h dark,light cycle) in crystallizing dishes filled with 100 ml of 50 μm pre-filtered surface seawater collected the same day at LTER-MC. After 24 h, the females were removed from the crystallizing dishes and the number of spawned and chewed eggs as well as the number of faecal pellets were counted under an inverted microscope (Zeiss Axiovert25) at 25x magnification [120].

The crystallizing dishes were incubated (20 °C, 12 h,12 h dark:light cycle) for additional 48 h to allow eggs to hatch. Subsequently, the number of dead nauplii (stage NI) laying on the bottom of the crystallizing dishes was counted under the inverted microscope, in order to assess survival of first non-feeding nauplii (NI), and thus, maternally-related larval survivorship. The content of the crystallizing dish was then fixed by adding 15 ml of ethanol (96%), and the number of hatched membranes, the non-viable eggs and total nauplii present in the container were counted. Hatching success of the eggs as well as naupliar survival rates were calculated following [40]:

$$ Hatching\ success=\frac{N\ (membranes)}{N\ \left( laid\ eggs\right)}\ 100 $$

$$ Naupliar\ survival=100-\left(\ \frac{N\ \left( dead\ nauplii\right)}{N\ (membranes)}\ 100\right) $$

Differences in faecal pellet and egg productions (N per female per day), egg hatching (%) and NI naupliar survival (%) between samples collected on May 23rd and 30th were analysed on the basis of t-tests considering Welch’s correction for unequal variances (*N* = 15). To avoid type I error, statistical significance was considered at 99%.

### RNA extraction and de novo transcriptome assembly

A number of 30–60 adult females of *T. stylifera* were sorted from the zooplankton sample collected on the 23rd and the 30th of May 2017 at LTER-MC and transferred to three replicate 1.5 ml Eppendorf tubes per date. In particular, two replicates from May 23rd consisted of 8 individuals, while one replicate contained 10 individuals. The three replicates from May 30th all consisted of 10 specimens. Samples were immediately frozen in liquid nitrogen after removing excess water and finally stored at − 80 °C for later total RNA extraction. These dates were selected because of the opposite naupliar survival rates measured in these two consecutive samples (25 and 93%, respectively; t-test considering unequal variances; *N* = 15; see results).

Total RNA from pools of *T. stylifera* females (8–10 individuals per pool), was extracted using the RNeasy Micro Kit (Qiagen, Hilden, Germany) [112], following manufacturer’s specification and performing on-column DNase-I treatment. The RNA was finally eluted in 15 μl of RNase-free water. RNA concentration (ng/μl) and purity were assessed through Nanodrop ND-1000 UV-Vis spectrophotometer (Marshall Scientific, Hampton, USA). Overall RNA integrity and DNA contamination were checked by electrophoresis of at least 200 ng of RNA on a 1% agarose gel in 0.5x Tris Borate EDTA buffer (TBE) and by analysing 150–200 ng of RNA in a 6000 Nano LabChip of an Agilent Bioanalyzer 2100 (Agilent Technologies, Santa Clara, USA), which defines RNA quality as RNA Integrity Number (RIN) [121]. RIN values > 8 were considered suitable for NGS analysis.

At least 2 μg of extracted RNA per sample (200 ng/μL) were delivered to Genomix4Life S.r.l. (Laboratory of Molecular Medicine and Genomics of the University of Salerno, Salerno, Italy) for library preparation, sequencing and de novo transcriptome assembly. Six cDNA libraries, each one generated from a single RNA sample extracted from groups of 8–10 individuals, were prepared using TruSeq RNA Sample Prep Kit (Illumina) according to manufacturer’s recommendations and pooled such that each index-tagged sample was present in equal-molar amounts. The pooled samples were subjected to cluster generation and multiplexed sequencing using an Illumina HiSeq 2500 platform (Illumina, San Diego, USA) in a 2 × 100 paired-end format. Raw reads were cleaned, trimmed and clipped with BBDuk (https://jgi.doe.gov/data-and-tools/bbtools/) setting a minimum phred score (Q) of 20 (base call accuracy of 99%), and a minimum length of 35 nucleotides. The quality of the reads before and after trimming was checked with the software FASTQC (http://www.bioinformatics.babraham.ac.uk/projects/fastqc/). High quality paired-end reads from all samples were used as input for transcriptome assembly using Trinity [122]. A filter for contaminants was performed by BLASTing the transcripts against the NCBI nr database, discarding all the sequences having a significant hit (E-value ≤0.0001) against bacteria or vegetal cells. Two different transcriptomes were generated, a ‘full’ transcriptome comprising all Trinity assembled transcripts (isoforms) and a ‘reference’ transcriptome of ‘Trinity predicted genes’, consisting of assembled transcripts with unique TR#_c#_g# identifiers (unigenes). This latter transcriptome contained either singletons (transcripts with a single isoform, ‘i’) as well as the longest isoform of transcripts having multiple ‘Trinity predicted isoforms’ (TR#_c#_g#_i#) [123].

### *Differential expression analysis and functional annotation of Temora stylifera transcriptome*

Quantification of de novo assembled transcripts (isoforms) and unigenes abundance for each sample was assessed using RSEM software provided by the Trinity package [124], after mapping back the reads on the ‘full’ and the ‘reference’ transcriptome, respectively, using STARS [125]. Mean expression levels of isoforms and unigenes read counts, expressed as Counts Per Million (CPM), were used as input to perform Differential Expression (DE) analysis between samples collected on May 23rd with respect to samples collected on May 30th, using the Trinity DeSeq2 package [126]. Statistical significance was obtained by performing a hypergeometric test and corrected *p*-value using the False Discovery Rate (FDR) method [127] and isoforms or unigenes having a FDR ≤0.05 were considered differentially expressed.

Functional annotation of *T. stylifera* ‘full’ (isoforms) and ‘reference’ (unigenes) transcriptomes was performed using the comprehensive bioinformatics tool Blast2Go [128, 129]. Sequence similarity was found using BLASTx function (E-value cut-off set to 1^− 3^) [130], which compares a nucleotide query sequence translated in all reading frames against a non-redundant protein sequence database (nr) used to find potential translation products of an unknown nucleotide sequence. Default parameters were selected, with a number of HITs equal to 1. Subsequently, Gene Ontology annotation was associated to mapped sequences and divided by the three GO terms: Biological Process (BP), Molecular Function (MF) and Cellular Component (CC).

Differences between samples from May 23rd and May 30th were highlighted through heat-maps based on log-transformed raw counts of differentially expressed unigenes and isoforms. Moreover, separate heat-maps were represented to highlight differences in *A5 Odorant Binding Protein* isoforms and in isoforms annotated as sequences involved in reproduction and cell development processes. Euclidean distance was calculated among variables, which were clustered on the basis of the ‘complete-linkage’ method. Cluster analysis and heat-map representation was performed using R 3.6.3 implemented in R-Studio.

### Transcriptome validation through RT-qPCR

To validate Illumina sequencing and differential expression results, six reference genes (RGs), previously optimized in *Calanus helgolandicus* [27, 28], were tested in *T. stylifera* through RT-qPCR to identify the most stable genes in samples used for transcriptome assembly. RGs were: *Actin* (*Act*), *Histone 3* (*Ist*), two ribosomal units (*18S* RNA and the ribosomal protein *S20*) and *Ubiquitin* (*Ubi*). Firstly, primer specificity and amplification efficiency of the six reference genes were assessed. Complementary DNA (cDNA) needed as template for RT-qPCR analysis was retro-transcribed from 1 μg of *T. stylifera* total RNA, extracted as described before, in a final volume of 20 μl using iScriptTM cDNA Synthesis Kit (Bio-Rad, Hercules, USA) following the manufacturer’s instructions. PCRs were performed on a GeneAmp PCR System 9700 (Applied Biosystems, Foster City, USA), with 2 μl of 10× PCR reaction buffer (Roche), 2 μl of 10 × 2 mM dNTPs (Roche), 0.8 μl of 5 U/μl Taq polymerase (Roche), 1 μl of 20 pmol/μl of each primer (forward and reverse), 1 μl of *T. stylifera* template cDNA and nuclease-free water to 20 μl of final volume. The PCR program consisted of a denaturation step at 95 °C for 3 min, 40 cycles at 95 °C for 30 s, 60 °C for 1 min and 72 °C for 30 s, and a final extension step at 72 °C for 7 min. Amplified PCR products were analysed by electrophoresis on 1.5% agarose gel in 0.5x TBE buffer to check proper length specificity of the cDNA products.

RT-qPCR reactions for primer amplification efficiency were then performed in a MicroAmp Optical 384-Well reaction plates (Applied Biosystem, Foster City, USA) with optical adhesive covers (Applied Biosystem, Foster City, USA), using a Viia7 Real Time PCR system (Applied Biosystem, Foster City, USA). The final PCR volume for each sample was 10 μl, with 5 μl of SensiFAST SYBR Green Master Mix (Meridian Inc., Cincinnati, USA), 1 μl of cDNA template and 4 μl (concentration of 0.7 pmol/μl) of each primer pair. All RT-qPCR reactions were carried out in triplicate to capture intra-assay variability. Three negative controls (consisting of 1 μl of water instead of the cDNA template) were considered for each primer pair. PCR conditions for all samples analysed were set as follows: 95 °C for 20 s, 40 cycles of 95 °C for 1 s, and 60 °C for 20 s. Dissociation protocol with a gradient (0.5 °C every 30 s) from 65 °C to 95 °C was also used to investigate the specificity of the primers and presence of primer dimers. Gene-specific amplification was confirmed by a single peak in the melting curve analysis. To quantify gene expression, primer amplification efficiencies were calculated through six serial dilutions of cDNA (1, 1:5, 1:10, 1:50, 1:100 and 1:500) for all primer pairs. The reference equation for efficiency calculation is E = 10^–1/slope^, where the slope is obtained from a standard curve between Ct values and the log_10_ of each dilution factor.

Stability of the six reference genes in *T. stylifera* samples used for transcriptome analysis (i.e. females collected on the 23rd and the 30th of May) was finally identified through RT-qPCR analyses as described above, using 1 μl of cDNA template (dilution 1:5) obtained by retro-transcription of the same total RNA employed for the Illumina sequencing. The most stable reference genes were then evaluated using RefFinder (http://leonxie.esy.es/RefFinder/) [60], a user-friendly web-based tool for evaluating and screening reference genes from extensive experimental datasets. Based on the rankings from each program, RefFinder assigns an appropriate weight to an individual gene and calculates the geometric mean of their weights for the overall final ranking. RGs *18S* and *Ubi* were used as reference genes for the analyses, because they were indicated as the most stable ones.

Once the most stable reference genes were identified, 9 unigenes and 3 isoforms were selected from the list of Differentially Expressed Genes (DEGs) generated by Illumina sequencing and subsequent DE analysis and used to validate differential expression results through RT-qPCR.

In particular, the unigenes *Putative Odorant Binding Protein A5* (*A5*), *cAMP-Responsive Element-Binding Protein-Like2* (*Crebl*), *Putative ATP-Dependent RNA Helicase* (*Me31b*), *Serine/Threonine Protein Phosphatase* (*Ppa2*), *Protein Obstructor E-Like* (*Obst*), *E3 Ubiquitin-Protein Ligase Arih1-Like* (*Arih1*), *Arylsulfatase B-Like* (*Arsb*), *Heat-Shock Protein 70* (*Hsp70*), *Very Low-Density Lipoprotein Receptor-Like* (*Vldlr*) and the isoforms *MOB Kinase Activator 1B* (*Mob1b*), *ATP-Dependent RNA Helicase Vasa-Like* (*Vasa*) and *Platelet-Activating Factor Homolog 2* (*Pafah*) were selected on the basis of fold-change between samples collected on the 23rd of May 2017 with respect to samples collected on the 30th of May 2017, E-value and similarity percentage (indicating how much of the query sequence corresponds to the reference one in public databases) reported after DE analysis and functional annotation of the transcriptome.

These selected sequences were then used to design specific forward and reverse primers through the online available platform Primer3web 4.1.0 (http://bioinfo.ut.ee/primer3/) and synthesized by Sigma-Aldrich (Merk, Germany). Desired primer size was set in the range of 18–24 bp (optimum 20) and melting temperature (Tm) between 59 and 61 °C (optimum 60 °C). Amplification products ranged between 110 and 240 bp. To verify primer specificity, PCRs were performed as described for RGs. Length of PCR product was verified through agarose gel electrophoresis. Subsequently, specificity and amplification efficiencies of the primers were further tested in RT-qPCR, using serial dilutions (1, 1:5, 1:10, 1:50, 1:100) of *T. stylifera* cDNA template. Settings of qPCR reactions were the same as described before. As for RGs, gene-specific amplification was confirmed by a single peak in the melting curve analysis.

To validate differential expression analysis results, cDNA obtained from *T. stylifera* samples used for transcriptome analysis was used as template (1:10 dilution) for RT-qPCR reactions. Relative expression levels of each target gene in the samples collected on the 23rd of May was compared to samples collected on the 30th of May through the Pfaffl equation [131], using the tool REST (Relative Expression Software Tool) [132]. Results were analysed based on the following equation, where the relative expression of a target gene is compared in a ‘test’ sample (copepod collected on the 23rd of May) versus a ‘control’ (copepod collected on the 30th of May), normalized to each reference gene [131]:

$$ Ratio=\frac{E{(Target)}^{\varDelta Ct\  target\left( Control- Sample\right)}}{E{(Reference)}^{\varDelta Ct\  reference\left( Control- Sample\right)}} $$

The E (target) is the RT-qPCR efficiency of target gene primers; E (ref) is the RT-qPCR efficiency of the reference gene primers; ΔCt_(target)_ is the Ct deviation of control-sample of the target gene transcript; ΔCt_(reference)_ is the Ct deviation of control-sample of reference gene transcript.

## Supplementary information

**Additional file 1 Table S1:** De novo transcriptome assembly of *Temora stylifera*. Number of reads counted in the forward and the reverse filament are shown along with the number of assembled transcripts and Trinity predicted genes (unigenes), transcripts with unique TR#_c#_g# identifiers. The latter list includes singletons as well as the longest isoform of each predicted gene. Average transcript length, median and N_50_ are also indicated.

**Additional file 2 Figure S1:** Blast2Go statistics output for *Temora stylifera* de novo reference transcriptome assembly (unigenes). Percentage distribution of E-value (0 < E < 1^− 3^) and sequence similarity percentage (30–100%) are displayed on the top of the figure. Bottom panel describes top 20 blast hit taxon groups; a subplot of the total hits is shown for clarity.

**Additional file 3 Table S2:** *Temora stylifera* differentially expressed isoforms that received functional annotation in Blast2Go. Trinity ID number with predicted gene and isoform identifiers, length (bp), log_2_-Fold-Change (log_2_-FC), adjusted *p*-value (p-adj) of statistical analysis (FDR) for each predicted genes, sequence description and functional annotation as provided by Blast2Go are shown. Sequences are ordered by p-adj values within each down-regulated (negative log2-FC) and up-regulated (positive log2-FC) isoforms.

**Additional file 4 Table S3:** List of Reference Genes (RGs) tested in *Temora stylifera*. Protein name, function, amplicon length (A_L_) in base pairs (bp), primer sequence and amplification efficiencies (%E) are shown.

## Data Availability

Raw reads are stored into the NCBI Sequence Read Archive database under accession number PRJNA632714.

## Abbreviations

HDoHE: Hydroxy docosahexaenoic acid
RIN: RNA integrity number
FDR: False discovery rate
GO: Gene ontology
Obp: Odorant binding protein
Obst-E: Protein obstructor E-like
Hsp70: Heat shock protein 70
Mob1b: Mob kinase activator 1B
Vasa: ATP-dependent RNA helicase vasa-like
Pafah: Platelet-activating factor homolog 2
Arih1: Ubiquitin-protein ligase Arih1-like
Arsb: Arylsulfatase B-like
Crebl: cAMP responsive element binding protein-like2
Ppa2: Serine/Threonine protein phosphatase
Mthfd: Methylene tetrahydrofolate dehydrogenase
Tnrc18: Trinucleotide repeat-containing gene 18
Dpt: Dermatopontin
Mael: Protein maelstrom
PIP: Phosphatidylinositol phosphate
Me31b: Putative ATP-dependent RNA helicase
Klhl12: Kelk 12
GAG: Glycosaminoglycans
YPP: Yolk protein precursors
PPIn: Phosphatidylinositide
LTER-MC: Long-term ecological research station MareChiara
DE: Differential expression
CMP: Counts per million
BP: Biological process
MF: Molecular function
CC: Cellular component
Ist: Histone-3
Ubi: Ubiquitin
Act: Actin
