# Supplementary material for: RNA-Seq and differential gene expression analysis in Temora stylifera copepod females with contrasting non-feeding nauplii survival rates: an environmental transcriptomics study
Source: BMC Genomics. 2020 Oct 6;21:693. doi: 10.1186/s12864-020-07112-w (PMC7541278; doi:10.1186/s12864-020-07112-w)
Supplement: Supplementary file 1 — Additional file 1 Table S1: De novo transcriptome assembly of Temora stylifera. Number of reads counted in the forward and the reverse filament are shown along with the number of assembled transcripts and Trinity predicted genes (unigenes), transcripts with unique TR#_c#_g# identifiers. The latter list includes singletons as well as the longest isoform of each predicted gene. Average transcript length, median and N50 are also indicated. [file 12864_2020_7112_MOESM1_ESM.docx]

Table S1. *De novo* transcriptome assembly of *Temora stylifera*. Number of reads counted in the forward and the reverse filament are shown along with the number of assembled transcripts and Trinity predicted genes (unigenes), transcripts with unique TR#_c#_g# identifiers. The latter list includes singletons as well as the longest isoform of each predicted gene. Average transcript length, median and N_50_ are also indicated.

| **Category** | | **Number/Length** |
| --- | --- | --- |
| *Number of Reads R1* | | 131,993,195 |
| *Number of Reads R2* | | 131,993,195 |
| *Number of Transcripts* | | 268,665 |
|  | Average Transcripts Length | 517.6 bp |
|  | Median Transcripts Length | 310 bp |
|  | N_50_ | 655 bp |
| *Number of Unigenes* | | 120,749 |
