# Supplementary material for: RNA-Seq and differential gene expression analysis in Temora stylifera copepod females with contrasting non-feeding nauplii survival rates: an environmental transcriptomics study
Source: BMC Genomics. 2020 Oct 6;21:693. doi: 10.1186/s12864-020-07112-w (PMC7541278; doi:10.1186/s12864-020-07112-w)
Supplement: Supplementary file 2 — Additional file 2 Figure S1: Blast2Go statistics output for Temora stylifera de novo reference transcriptome assembly (unigenes). Percentage distribution of E-value (0 < E < 1− 3) and sequence similarity percentage (30–100%) are displayed on the top of the figure. Bottom panel describes top 20 blast hit taxon groups; a subplot of the total hits is shown for clarity. [file 12864_2020_7112_MOESM2_ESM.docx]

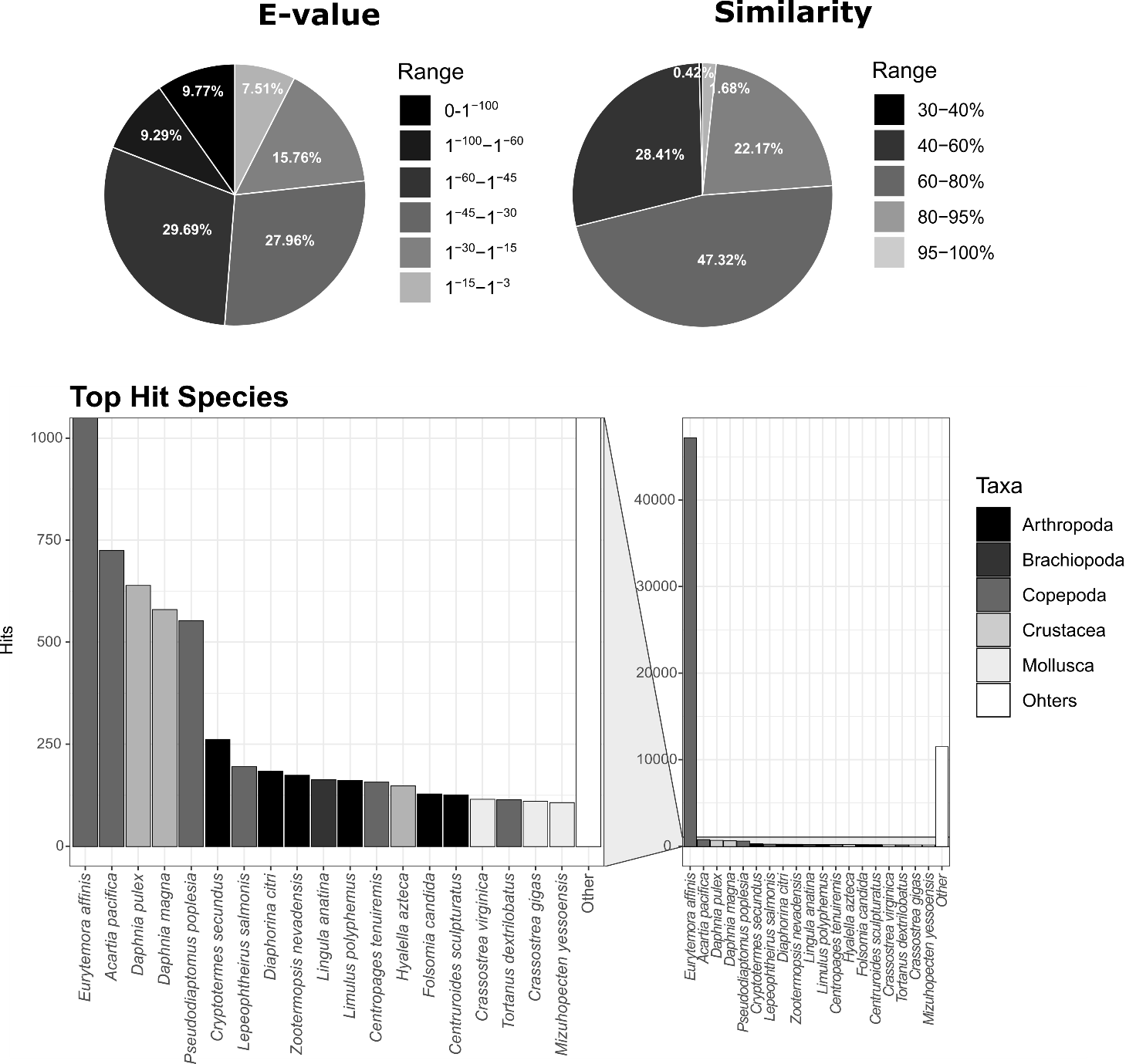


Figure S1. Blast2Go statistics output for *Temora stylifera de novo* transcriptome assembly. Percentage distribution of E-value (0<E<1^-3^) and sequence similarity percentage (30-100%) are displayed on the top of the figure. Bottom panel describes top 20 blast hit taxon groups; a subplot of the total hits is shown for clarity.
