# Supplementary material for: RNA-Seq and differential gene expression analysis in Temora stylifera copepod females with contrasting non-feeding nauplii survival rates: an environmental transcriptomics study
Source: BMC Genomics. 2020 Oct 6;21:693. doi: 10.1186/s12864-020-07112-w (PMC7541278; doi:10.1186/s12864-020-07112-w)
Supplement: Supplementary file 3 — Additional file 3 Table S2: Temora stylifera differentially expressed isoforms that received functional annotation in Blast2Go. Trinity ID number with predicted gene and isoform identifiers, length (bp), log2-Fold-Change (log2-FC), adjusted p-value (p-adj) of statistical analysis (FDR) for each predicted genes, sequence description and functional annotation as provided by Blast2Go are shown. Sequences are ordered by p-adj values within each down-regulated (negative log2-FC) and up-regulated (positive log2-FC) isoforms. [file 12864_2020_7112_MOESM3_ESM.docx]

Table S2. *Temora stylifera* differentially expressed isoforms that received functional annotation in Blast2Go. Trinity ID number with predicted gene and isoform identifiers, length (bp), log_2_-Fold-Change (log_2_-FC), adjusted p-value (p-adj) of statistical analysis (FDR) for each predicted genes, sequence description and functional annotation as provided by Blast2Go are shown. Sequences are ordered by p-adj values within each down-regulated (negative log_2_-FC) and up-regulated (positive log_2_-FC) isoforms.

| ***Sequence Name*** | ***Length (bp)*** | ***log_2_-FC*** | ***p-adj*** | ***Description*** | ***GO IDs*** |
| --- | --- | --- | --- | --- | --- |
| TRINITY_DN61915_c1_g1_i2 | 5754 | -10.34 | 9.55E-12 | 1-phosphatidylinositol 3-phosphate 5-kinase-like | GO:0004930;GO:0007186;GO:0016021 |
| TRINITY_DN62889_c0_g1_i1 | 7245 | -9.72 | 6.51E-11 | zinc finger SWIM domain-containing protein 8-like | GO:0008270 |
| TRINITY_DN56306_c0_g1_i1 | 279 | -4.75 | 9.98E-11 | OV-16 antigen-like | GO:0016021;GO:0022857;GO:0055085 |
| TRINITY_DN52078_c1_g1_i5 | 1160 | -9.48 | 4.26E-10 | MOB kinase activator 1B | GO:0004553;GO:0005576;GO:0005975;GO:0006030;GO:0008061 |
| TRINITY_DN58691_c0_g1_i4 | 1697 | -9.63 | 9.83E-10 | BRCA2-interacting transcriptional repressor EMSY-like | GO:0005743;GO:0006118;GO:0009055;GO:0016021;GO:0022904;GO:0046872;GO:0070469 |
| TRINITY_DN61508_c0_g1_i2 | 4149 | -9.72 | 3.24E-09 | uncharacterized protein Dere_GG13897, isoform C | GO:0003824;GO:0008152 |
| TRINITY_DN61841_c6_g1_i4 | 1233 | -9.29 | 1.73E-08 | protein trapped in endoderm-1-like | GO:0000159;GO:0005488;GO:0007165;GO:0016020;GO:0019888 |
| TRINITY_DN61595_c2_g1_i1 | 2369 | -9.39 | 1.01E-07 | serine/threonine-protein phosphatase 2A 56 kDa regulatory subunit gamma isoform-like isoform X1 | GO:0005515;GO:0005886;GO:0007275;GO:0044425 |
| TRINITY_DN59703_c2_g1_i1 | 254 | -2.56 | 2.56E-07 | putative odorant-binding protein A5 | GO:0000184;GO:0003676;GO:0004004;GO:0005515;GO:0005524;GO:0005730;GO:0006364;GO:0010501;GO:0071042;GO:1990120 |
| TRINITY_DN47549_c0_g2_i1 | 1414 | -8.48 | 7.40E-07 | cytosolic small ribosomal subunit | GO:0002181;GO:0003735;GO:0022627;GO:0042254 |
| TRINITY_DN52432_c2_g1_i3 | 223 | -5.23 | 1.93E-06 | uncharacterized protein LOC111718103 | GO:0000785;GO:0003682;GO:0030154 |
| TRINITY_DN51709_c0_g1_i5 | 2201 | -8.38 | 2.72E-06 | SET and MYND domain-containing protein 4-like | GO:0005515 |
| TRINITY_DN44995_c9_g1_i6 | 227 | -5.44 | 5.75E-06 | ferritin heavy chain A-like | GO:0004322;GO:0005623;GO:0006826;GO:0006879;GO:0008199;GO:0015994;GO:0051536;GO:0055114 |
| TRINITY_DN59824_c0_g1_i9 | 1448 | -7.91 | 9.86E-06 | uncharacterized protein LOC111704961 | GO:0005215;GO:0005515;GO:0016020;GO:0055085 |
| TRINITY_DN51438_c1_g2_i4 | 346 | -8.09 | 1.97E-05 | 14-3-3zeta, partial | GO:0019904 |
| TRINITY_DN51778_c4_g1_i12 | 285 | -4.89 | 2.49E-05 | putative odorant-binding protein A5 | GO:0005515 |
| TRINITY_DN63585_c4_g1_i2 | 245 | -2.73 | 5.65E-05 | cytochrome oxidase subunit 1 (mitochondrion) | GO:0004129;GO:0005743;GO:0006123;GO:0016021;GO:0020037;GO:0045277;GO:0046872;GO:1902600 |
| TRINITY_DN54322_c1_g1_i1 | 202 | -5.18 | 8.41E-05 | OV-16 antigen-like | GO:0008152;GO:0016787 |
| TRINITY_DN48819_c0_g1_i1 | 1110 | -1.94 | 1.05E-04 | protein binding | GO:0005515 |
| TRINITY_DN61324_c6_g3_i3 | 552 | -1.28 | 1.43E-04 | arylsulfatase B-like | GO:0008152;GO:0008484 |
| TRINITY_DN56650_c0_g2_i1 | 2126 | -7.73 | 1.58E-04 | uncharacterized protein LOC111708691 | GO:0005515 |
| TRINITY_DN51429_c3_g1_i3 | 530 | -8.03 | 1.69E-04 | transmembrane transport | GO:0016021;GO:0055085 |
| TRINITY_DN50660_c0_g1_i2 | 1041 | -2.35 | 1.69E-04 | putative odorant-binding protein A5 | GO:0003676 |
| TRINITY_DN59576_c0_g1_i1 | 1031 | -8.17 | 2.95E-04 | ATP-dependent RNA helicase p62-like | GO:0003333;GO:0015171 |
| TRINITY_DN46142_c0_g1_i1 | 228 | -1.63 | 2.96E-04 | protein obstructor-E-like | GO:0005576;GO:0006030;GO:0008061 |
| TRINITY_DN45761_c1_g1_i4 | 232 | -3.39 | 6.39E-04 | cytochrome oxidase subunit I (mitochondrion) | GO:0004129;GO:0005743;GO:0006123;GO:0016021;GO:0020037;GO:0045277;GO:0046872;GO:1902600 |
| TRINITY_DN58090_c0_g1_i4 | 515 | -7.34 | 8.17E-04 | hypothetical protein CERSUDRAFT_54156 | GO:0006120;GO:0006744;GO:0006814;GO:0008137;GO:0015992 |
| TRINITY_DN63868_c1_g1_i2 | 16797 | -9.59 | 1.15E-03 | 16797 | GO:0003676 |
| TRINITY_DN53590_c0_g1_i1 | 784 | -7.67 | 1.41E-03 | RNA 3'-terminal phosphate cyclase-like | GO:0004129;GO:0005743;GO:0006123;GO:0016021;GO:0020037;GO:0045277;GO:0046872;GO:1902600 |
| TRINITY_DN50801_c1_g2_i4 | 1251 | -6.96 | 1.85E-03 | uncharacterized protein LOC111699566 | GO:0016757 |
| TRINITY_DN56133_c1_g4_i1 | 303 | -2.11 | 4.30E-03 | sequestosome-1-like | GO:0005328;GO:0006812;GO:0006836;GO:0016021 |
| TRINITY_DN43307_c0_g1_i2 | 1231 | -6.79 | 4.86E-03 | uncharacterized protein LOC111703668 isoform X1 | GO:0004252;GO:0006508 |
| TRINITY_DN35563_c0_g1_i8 | 388 | -6.64 | 5.31E-03 | collagen alpha-1(V) chain-like | GO:0004222;GO:0005201;GO:0005578;GO:0005581;GO:0006508;GO:0046872 |
| TRINITY_DN48041_c0_g2_i3 | 207 | -1.98 | 6.10E-03 | hypothetical protein BA059_22125 | GO:0004099;GO:0005576;GO:0005975;GO:0006030;GO:0007632;GO:0008061;GO:0035001;GO:0035159;GO:0060439 |
| TRINITY_DN44787_c0_g1_i11 | 235 | -5.75 | 7.28E-03 | AF484038_1cytochrome oxidase subunit II (mitochondrion) | GO:0004129;GO:0005507;GO:0005739;GO:0006123;GO:0015992;GO:0045277 |
| TRINITY_DN57113_c0_g1_i2 | 1218 | -6.55 | 7.54E-03 | dermatopontin-like isoform X1 | GO:0003743;GO:0005515;GO:0005840;GO:0006446 |
| TRINITY_DN46002_c0_g1_i1 | 370 | -2.04 | 8.15E-03 | cAMP-responsive element-binding protein-like 2 | GO:0003700;GO:0005667;GO:0006355 |
| TRINITY_DN45761_c1_g1_i10 | 205 | -3.65 | 9.57E-03 | cytochrome oxidase subunit I (mitochondrion) | GO:0004129;GO:0005743;GO:0006123;GO:0016021;GO:0020037;GO:0045277;GO:0046872;GO:1902600 |
| TRINITY_DN54699_c0_g1_i3 | 3513 | -6.45 | 9.62E-03 | cAMP-specific 3',5'-cyclic phosphodiesterase 4C-like isoform X1 | GO:0003678;GO:0005524;GO:0005657;GO:0006260 |
| TRINITY_DN55897_c0_g1_i1 | 2619 | -1.38 | 1.18E-02 | sodium-dependent nutrient amino acid transporter 1-like | GO:0005975;GO:0016301 |
| TRINITY_DN52028_c0_g1_i1 | 1549 | -0.71 | 1.31E-02 | chitinase-3-like protein 1 | GO:0015031;GO:0030176 |
| TRINITY_DN56485_c0_g1_i1 | 1864 | -2.08 | 1.36E-02 | innexin inx2-like isoform X1 | GO:0003847;GO:0008247;GO:0016042;GO:0046486 |
| TRINITY_DN58044_c3_g1_i2 | 344 | -4.70 | 1.43E-02 | NADH dehydrogenase subunit 4 (mitochondrion) |  |
| TRINITY_DN59770_c0_g1_i3 | 2680 | -1.00 | 1.62E-02 | solute carrier organic anion transporter family member 5A1-like | GO:0046872 |
| TRINITY_DN47394_c2_g1_i3 | 219 | -1.14 | 1.69E-02 | iron ion binding | GO:0005506 |
| TRINITY_DN46372_c1_g2_i11 | 248 | -1.49 | 1.80E-02 | 40S ribosomal protein S11 | GO:0003735;GO:0005840;GO:0006412;GO:0042254 |
| TRINITY_DN57136_c0_g1_i3 | 2773 | -2.07 | 1.95E-02 | uncharacterized protein LOC111709168 | GO:0005576;GO:0009405 |
| TRINITY_DN55139_c3_g1_i1 | 281 | -1.48 | 2.18E-02 | transposase activity | GO:0004803;GO:0006313 |
| TRINITY_DN56639_c0_g1_i2 | 3248 | -1.06 | 2.18E-02 | protein unc-45 homolog B-like | GO:0000922;GO:0005524;GO:0005737;GO:0005813;GO:0005874;GO:0007049;GO:0008017;GO:0008568;GO:0045298;GO:0051013;GO:0051301 |
| TRINITY_DN58880_c2_g3_i1 | 574 | -0.84 | 2.83E-02 | uncharacterized protein LOC111701982 | GO:0003824;GO:0046872 |
| TRINITY_DN45964_c0_g1_i1 | 1180 | -6.56 | 2.94E-02 | carbohydrate sulfotransferase 11-like isoform X1 | GO:0008146;GO:0016021;GO:0016051 |
| TRINITY_DN58203_c2_g2_i18 | 1141 | -4.46 | 3.26E-02 | TPA: hypothetical protein | GO:0004672;GO:0005524;GO:0005856;GO:0006468 |
| TRINITY_DN44989_c1_g1_i2 | 221 | -4.34 | 3.26E-02 | 60S ribosomal protein L27 | GO:0003735;GO:0006412;GO:0022625;GO:0042254;GO:0098556 |
| TRINITY_DN46836_c0_g1_i7 | 202 | -1.13 | 3.48E-02 | ribosomal protein S11 |  |
| TRINITY_DN58848_c2_g5_i3 | 350 | -1.11 | 3.56E-02 | cytochrome b-c1 complex subunit 2, mitochondrial-like | GO:0003676;GO:0006259 |
| TRINITY_DN46430_c2_g2_i2 | 627 | -1.27 | 3.71E-02 | heat-shock protein Hsp70 | GO:0005524 |
| TRINITY_DN53513_c3_g1_i22 | 234 | -1.33 | 4.10E-02 | cytochrome c oxidase subunit I (mitochondrion) | GO:0004129;GO:0005743;GO:0006123;GO:0016021;GO:0020037;GO:0045277;GO:0046872;GO:1902600 |
| TRINITY_DN53513_c3_g1_i1 | 214 | -3.10 | 4.15E-02 | cytochrome oxidase subunit I (mitochondrion) |  |
| TRINITY_DN46292_c1_g1_i6 | 290 | -4.47 | 4.31E-02 | 60S ribosomal protein L26-like | GO:0002181;GO:0003723;GO:0003735;GO:0022625;GO:0042273 |
| TRINITY_DN54808_c0_g1_i1 | 1258 | -1.11 | 4.37E-02 | arginine kinase-like | GO:0004114;GO:0006144;GO:0007165 |
| TRINITY_DN58726_c2_g1_i12 | 399 | -6.00 | 4.80E-02 | uncharacterized protein LOC111717719 | GO:0016021 |
| TRINITY_DN46306_c0_g1_i7 | 316 | 9.84 | 4.82E-11 | ADP,ATP carrier protein 3 | GO:0005743;GO:0016021;GO:0022857;GO:0055085 |
| TRINITY_DN51343_c0_g1_i5 | 2445 | 8.76 | 1.54E-08 | monocarboxylate transporter 2-like | GO:0016021;GO:0055085 |
| TRINITY_DN63022_c3_g1_i6 | 2776 | 9.52 | 3.26E-08 | tyrosine-protein phosphatase non-receptor type 9-like | GO:0004725;GO:0006470;GO:0006570 |
| TRINITY_DN49892_c0_g1_i2 | 1649 | 8.46 | 1.01E-07 | stomatin-4-like isoform X2 | GO:0016021 |
| TRINITY_DN52728_c0_g1_i13 | 3434 | 8.32 | 2.54E-07 | mitotic cytokinesis | GO:0000281;GO:0000776;GO:0005515;GO:0005635;GO:0005737;GO:0005813;GO:0008045;GO:0009792;GO:0016301;GO:0016310;GO:0030334;GO:0030426;GO:0033365;GO:0040038;GO:0044297;GO:0050832;GO:0051304 |
| TRINITY_DN62990_c0_g1_i1 | 6253 | 8.40 | 4.89E-07 | protein TANC2 isoform X1 | GO:0005515 |
| TRINITY_DN63480_c0_g1_i1 | 6707 | 8.41 | 6.12E-07 | serine/threonine-protein kinase SMG1-like | GO:0005515;GO:0016301 |
| TRINITY_DN62038_c7_g1_i2 | 216 | 7.37 | 3.19E-06 | heat shock 70 kDa protein cognate 4-like | GO:0005524;GO:0016307;GO:0035556;GO:0046488;GO:0046872 |
| TRINITY_DN48585_c5_g2_i3 | 242 | 1.01 | 4.61E-06 | mitochondrial respiratory chain complex III | GO:0005750;GO:0006118;GO:0006122;GO:0020037;GO:0045153;GO:0046872 |
| TRINITY_DN57759_c4_g3_i1 | 224 | 3.11 | 1.92E-05 | DNA-binding transcription factor | GO:0003700;GO:0005634;GO:0005667;GO:0006355;GO:0043565 |
| TRINITY_DN44685_c4_g1_i4 | 647 | 7.82 | 2.09E-05 | tubulin beta-2C chain | GO:0005200;GO:0005525;GO:0005737;GO:0005874;GO:0007010;GO:0007017 |
| TRINITY_DN59539_c0_g3_i2 | 2221 | 2.33 | 6.55E-05 | sodium-coupled neutral amino acid transporter 9-like | GO:0005515;GO:0005938;GO:0007280;GO:0007315;GO:0007472;GO:0008104;GO:0031466;GO:0035017;GO:0045495;GO:0045732;GO:0046843;GO:0048471;GO:0070449 |
| TRINITY_DN63479_c1_g1_i3 | 2921 | 8.36 | 1.04E-04 | isoleucine--tRNA ligase, mitochondrial-like | GO:0002161;GO:0004822;GO:0005524;GO:0005737;GO:0006428;GO:0009097;GO:0009098;GO:0009099 |
| TRINITY_DN46736_c4_g1_i3 | 201 | 1.30 | 1.69E-04 | Hypothetical protein, conserved | GO:0005515;GO:0005840 |
| TRINITY_DN59211_c1_g1_i1 | 1470 | 8.09 | 2.08E-04 | glutamyl aminopeptidase-like isoform X2 | GO:0060964 |
| TRINITY_DN53126_c0_g2_i1 | 2357 | 7.45 | 2.31E-04 | zinc finger and BTB domain-containing protein 14-like isoform X2 | GO:0020037;GO:0046872 |
| TRINITY_DN42960_c0_g2_i5 | 1520 | 7.14 | 3.37E-04 | ATP-dependent RNA helicase vasa-like | GO:0003676;GO:0005524;GO:0016787 |
| TRINITY_DN54042_c0_g1_i1 | 2084 | 3.97 | 3.39E-04 | uncharacterized protein LOC111241867 | GO:0003676 |
| TRINITY_DN54686_c2_g1_i2 | 2749 | 5.02 | 6.25E-04 | twinkle protein, mitochondrial-like | GO:0009987 |
| TRINITY_DN57354_c2_g2_i1 | 1242 | 7.03 | 7.46E-04 | protein phosphatase 1H-like | GO:0016614;GO:0050660;GO:0055114 |
| TRINITY_DN52500_c0_g1_i8 | 233 | 5.09 | 9.35E-04 | G-protein comlpex beta subunit CpcB | GO:0016021;GO:0055085 |
| TRINITY_DN57615_c1_g1_i7 | 1210 | 7.42 | 1.58E-03 | uncharacterized protein LOC111709725 | GO:0004722;GO:0006470;GO:0008287;GO:0046872 |
| TRINITY_DN50969_c0_g3_i2 | 787 | 6.88 | 1.63E-03 | uncharacterized protein LOC111702633 isoform X1 | GO:0016757 |
| TRINITY_DN57108_c1_g2_i7 | 1119 | 8.03 | 1.63E-03 | transcription initiation factor TFIID subunit 5-like | GO:0008152;GO:0008484 |
| TRINITY_DN54182_c0_g1_i1 | 1372 | 7.00 | 1.83E-03 | uncharacterized protein LOC110459190 | GO:0005515 |
| TRINITY_DN60920_c2_g1_i3 | 1189 | 6.83 | 2.74E-03 | autotransporter outer membrane beta-barrel domain-containing protein | GO:0005905;GO:0006886;GO:0030121;GO:0035615;GO:0072583 |
| TRINITY_DN59517_c0_g1_i1 | 944 | 4.35 | 4.19E-03 | uncharacterized protein Dvir_GJ21895, isoform A | GO:0006508;GO:0016787 |
| TRINITY_DN55018_c3_g2_i7 | 1901 | 6.62 | 4.86E-03 | Transposable element P transposase | GO:0006030;GO:0008061 |
| TRINITY_DN47526_c2_g1_i11 | 410 | 5.22 | 5.41E-03 | 40S ribosomal protein S3a |  |
| TRINITY_DN60491_c0_g1_i10 | 1009 | 6.92 | 5.50E-03 | phosphorylated adapter RNA export protein-like | GO:0005515;GO:0005634;GO:0044427 |
| TRINITY_DN45315_c0_g1_i1 | 2859 | 4.45 | 5.71E-03 | hypothetical protein BOX15_Mlig011095g2, partial | GO:0004488;GO:0009396;GO:0016742;GO:0044238;GO:0046487;GO:0055114 |
| TRINITY_DN53791_c1_g1_i6 | 250 | 2.14 | 5.96E-03 | protein binding | GO:0005515;GO:0006491 |
| TRINITY_DN57863_c8_g1_i3 | 330 | 1.59 | 6.83E-03 | nucleic acid binding | GO:0003676;GO:0005515;GO:0006508;GO:0016787;GO:0044267 |
| TRINITY_DN62476_c3_g1_i7 | 2118 | 6.82 | 6.83E-03 | serine/arginine repetitive matrix protein 2-like | GO:0005515 |
| TRINITY_DN51440_c1_g1_i6 | 279 | 1.22 | 7.41E-03 | protein domain specific binding | GO:0019904 |
| TRINITY_DN55259_c0_g2_i2 | 1207 | 7.03 | 7.99E-03 | dipeptidase 1-like | GO:0005506;GO:0016705;GO:0020037;GO:0055114 |
| TRINITY_DN55457_c0_g1_i1 | 1735 | 7.31 | 8.35E-03 | putative neutral sphingomyelinase | GO:0006508;GO:0008237;GO:0016805;GO:0031224 |
| TRINITY_DN63004_c3_g1_i9 | 2924 | 6.59 | 1.09E-02 | uncharacterized protein LOC111699347 | GO:0016020 |
| TRINITY_DN55207_c2_g1_i4 | 2179 | 6.88 | 1.09E-02 | cytochrome P450 3080A1 |  |
| TRINITY_DN52997_c0_g1_i9 | 1747 | 6.67 | 1.12E-02 | uncharacterized protein LOC111712824 isoform X2 | GO:0006751;GO:0036374 |
| TRINITY_DN59759_c0_g1_i7 | 1717 | 0.98 | 1.16E-02 | collagen alpha-1(I) chain-like isoform X2 | GO:0005515 |
| TRINITY_DN63486_c4_g1_i3 | 4453 | 7.71 | 1.36E-02 | phosphatidate phosphatase LPIN2-like isoform X1 | GO:0005515;GO:0005634;GO:0005737;GO:0007474;GO:0019217;GO:0019432;GO:0030514;GO:0035183;GO:0042594;GO:0055088 |
| TRINITY_DN54885_c2_g2_i15 | 2361 | 6.44 | 1.47E-02 | uncharacterized protein LOC111711709 isoform X1 | GO:0004054;GO:0005524;GO:0006525;GO:0006560;GO:0016310 |
| TRINITY_DN62176_c0_g1_i4 | 2164 | 6.84 | 1.54E-02 | uncharacterized protein LOC111700460 | GO:0005524;GO:0032440;GO:0055114 |
| TRINITY_DN58828_c12_g2_i2 | 770 | 1.16 | 1.62E-02 | replication protein A 70 kDa DNA-binding subunit-like | GO:0019284;GO:0019509;GO:0046523 |
| TRINITY_DN46903_c1_g1_i9 | 259 | 1.25 | 1.68E-02 | helix-turn-helix domain-containing protein | GO:0003735;GO:0005840;GO:0006412;GO:0042254 |
| TRINITY_DN49384_c5_g1_i3 | 295 | 2.84 | 1.80E-02 | hypothetical protein DDG59_07555 | GO:0005515 |
| TRINITY_DN62627_c7_g1_i1 | 378 | 4.60 | 1.80E-02 | protein binding | GO:0005515 |
| TRINITY_DN53649_c0_g1_i1 | 900 | 6.33 | 2.09E-02 | glucosidase 2 subunit beta-like | GO:0000166;GO:0003963;GO:0005634;GO:0006396 |
| TRINITY_DN52232_c0_g1_i1 | 2680 | 6.28 | 2.18E-02 | phospholipase D alpha 1-like | GO:0016301;GO:0016310 |
| TRINITY_DN50261_c1_g1_i2 | 237 | 1.72 | 2.91E-02 | membrane protein | GO:0016020 |
| TRINITY_DN58138_c0_g4_i3 | 3106 | 6.53 | 3.10E-02 | focal adhesion kinase 1-like isoform X2 | GO:0005515 |
| TRINITY_DN56524_c2_g4_i1 | 1318 | 1.70 | 3.33E-02 | katanin p60 ATPase-containing subunit A-like 1 | GO:0005921 |
| TRINITY_DN59104_c0_g2_i1 | 976 | 6.22 | 3.91E-02 | protein maelstrom 2-like isoform X1 | GO:0016021;GO:0022857;GO:0055085 |
| TRINITY_DN44116_c1_g1_i1 | 1536 | 3.77 | 4.67E-02 | ligand-gated ion channel 4-like precursor | GO:0004888;GO:0005230;GO:0007165;GO:0016021;GO:0034220 |
