# Supplementary material for: RNA-Seq and differential gene expression analysis in Temora stylifera copepod females with contrasting non-feeding nauplii survival rates: an environmental transcriptomics study
Source: BMC Genomics. 2020 Oct 6;21:693. doi: 10.1186/s12864-020-07112-w (PMC7541278; doi:10.1186/s12864-020-07112-w)
Supplement: Supplementary file 4 — Additional file 4 Table S3: List of Reference Genes (RGs) tested in Temora stylifera. Protein name, function, amplicon length (AL) in base pairs (bp), primer sequence and amplification efficiencies (%E) are shown. [file 12864_2020_7112_MOESM4_ESM.docx]

Table S3. List of Reference Genes (RGs) tested in *Temora stylifera*. Protein name, function, amplicon length (A_L_) in base pairs (bp), primer sequence and amplification efficiency percentages (%E) are shown.

| **Name** | **Function** | **A_L_ (bp)** |  | **Primers** | **%E** |
| --- | --- | --- | --- | --- | --- |
| *Actin* | Cytoskeleton structure | 128 | F | GGCACCACACTTTCTACAACG | 90 |
|  |  |  | R | GTTGAAGGTCTCGAACATGATC |  |
| *Histone3* | Chromatin structure | 137 | F | GAGGAGTGAAGAAGCCCCAC | 93 |
|  |  |  | R | TGAAGTCCTGAGCAATCTCCC |  |
| *18S* | Ribosome unit | 164 | F | GAAACCAAAGCATTTGGGTTC | 98 |
|  |  |  | R | GCTATCAATCTGTCAATCCTTCC |  |
| *S20* | Ribosome unit | 113 | F | CGTAAGACTCCTTGTGGTGAGG | 97 |
|  |  |  | R | GAAGTGATCTGCTTCACGATCTC |  |
| *Ubiquitin* | Protein degradation | 113 | F | GCAAGACCATCACCCTTGAG | 99 |
|  |  |  | R | CAGCGAAAGATCAACCTCTG |  |
